# Structure-function analysis of fission yeast cleavage and polyadenylation factor (CPF) subunit Ppn1 and its interactions with Dis2 and Swd22

**DOI:** 10.1371/journal.pgen.1009452

**Published:** 2021-03-12

**Authors:** Bradley Benjamin, Ana M. Sanchez, Angad Garg, Beate Schwer, Stewart Shuman

**Affiliations:** 1 Molecular Biology Program, Sloan-Kettering Institute, New York, New York, United States of America; 2 Gerstner Sloan Kettering Graduate School of Biomedical Sciences, New York, New York, United States of America; 3 Dept. of Microbiology and Immunology, Weill Cornell Medical College, New York, New York, United States of America; The University of North Carolina at Chapel Hill, UNITED STATES

## Abstract

Fission yeast Cleavage and Polyadenylation Factor (CPF), a 13-subunit complex, executes the cotranscriptional 3’ processing of RNA polymerase II (Pol2) transcripts that precedes transcription termination. The three-subunit DPS sub-complex of CPF, consisting of a PP1-type phosphoprotein phosphatase Dis2, a WD-repeat protein Swd22, and a putative phosphatase regulatory factor Ppn1, associates with the CPF core to form the holo-CPF assembly. Here we probed the functional, physical, and genetic interactions of DPS by focusing on the Ppn1 subunit, which mediates association of DPS with the core. Transcriptional profiling by RNA-seq defined limited but highly concordant sets of protein-coding genes that were dysregulated in *ppn1*Δ, *swd22*Δ and *dis2*Δ cells, which included the *DPS*Δ down-regulated phosphate homeostasis genes *pho1* and *pho84* that are controlled by lncRNA-mediated transcriptional interference. Essential and inessential modules of the 710-aa Ppn1 protein were defined by testing the effects of Ppn1 truncations in multiple genetic backgrounds in which Ppn1 is required for growth. An N-terminal 172-aa disordered region was dispensable and its deletion alleviated hypomorphic phenotypes caused by deleting C-terminal aa 640–710. A TFIIS-like domain (aa 173–330) was not required for viability but was important for Ppn1 activity in phosphate homeostasis. Distinct sites within Ppn1 for binding to Dis2 (spanning Ppn1 aa 506 to 532) and Swd22 (from Ppn1 aa 533 to 578) were demarcated by yeast two-hybrid assays. Dis2 interaction-defective missense mutants of full-length Ppn1 (that retained Swd22 interaction) were employed to show that binding to Dis2 (or its paralog Sds21) was necessary for Ppn1 biological activity. Ppn1 function was severely compromised by missense mutations that selectively affected its binding to Swd22.

## Author summary

Multi-layered regulatory inputs to eukaryal gene expression target the initiation, elongation, and termination steps of RNA polymerase II transcription. Termination is coupled to, and ensues from, the 3’ processing of nascent RNA by multi-protein assemblies. The fission yeast Cleavage and Polyadenylation Factor complex, CPF, is an exemplary 3’ processing machine composed of 13 protein subunits, three of which (named Dis2, Ppn1, and Swd22) form a DPS sub-complex within CPF. In this study, we interrogated the effects of DPS null mutations on the fission yeast transcriptome. We probed the physical interactions of Ppn1 and its genetic interactions with other components of the transcription and RNA processing machinery. We delineated distinct binding sites within Ppn1 for Dis2 and Swd22 and then showed that perturbation of these sites interdicts Ppn1’s essential activities in vivo.

## Introduction

**Fig 1:**
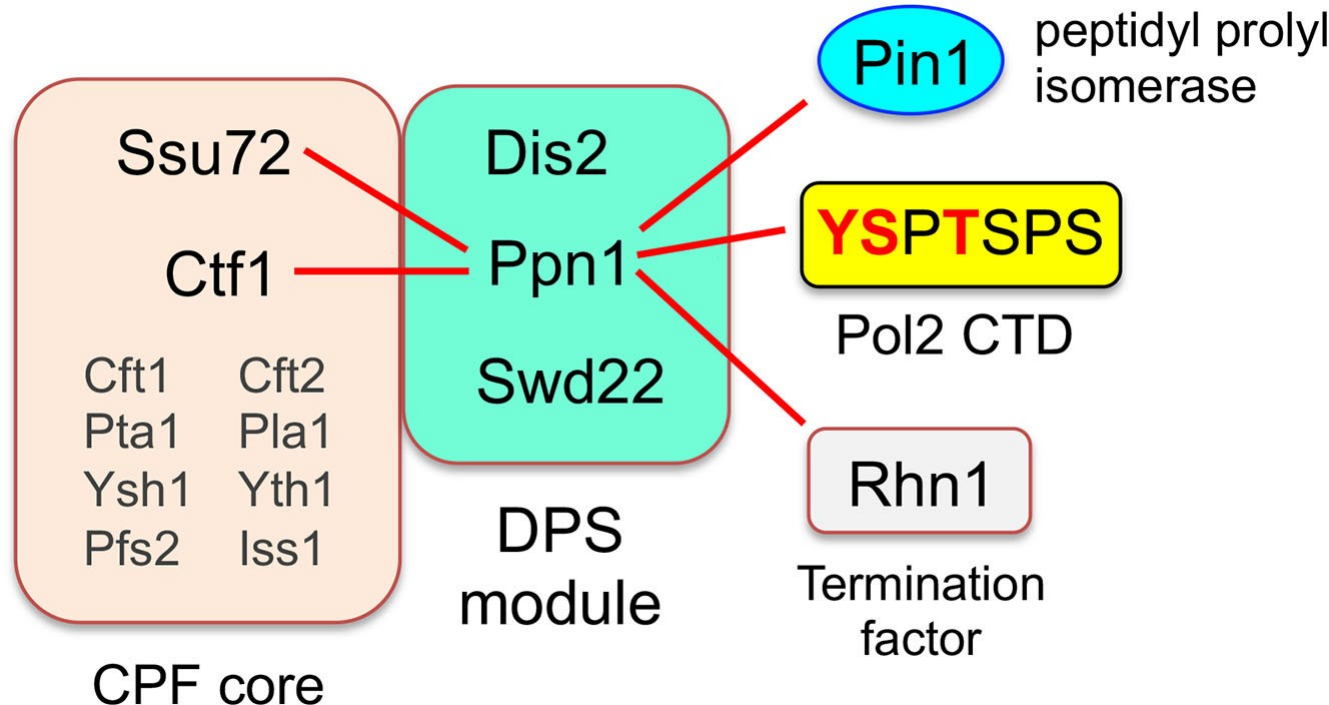
Organization of fission yeast CPF and genetic interactions of its subunits. Essential subunits of the CPF core are shown in gray font; inessential subunits of the core and DPS module are in black font. Synthetic lethalities of *ppn1*Δ with mutant alleles of CPF subunits, termination factor Rhn1, Pol2 CTD phospho-site mutants (red font), and prolyl isomerase Pin1 are denoted by red lines.

Fission yeast *Schizosaccharomyces pombe* Cleavage and Polyadenylation Factor (CPF) is a 13-subunit protein assembly responsible for the cotranscriptional 3’ processing of RNA polymerase II (Pol2) transcripts that precedes Pol2 transcription termination [1]. Holo-CPF consists of two component complexes (Fig 1): a 10-subunit CPF core composed of proteins Ysh1 (the cleavage endonuclease), Pla1 (the poly(A) polymerase), Pta1, Yth1 (a zinc finger protein), Pfs2 (a WD repeat protein), Iss1, Cft1 (a WD repeat protein), Cft2 (a metallo-β-lactamase/β-CASP protein), Ctf1, and Ssu72 (a phosphoprotein phosphatase); and a 3-subunit DPS complex comprising Dis2 (a phosphoprotein phosphatase), Ppn1, and Swd22 (a WD repeat protein). Eight of the CPF core subunits, including the cleavage endonuclease and poly(A) polymerase, are essential for fission yeast viability. Two of the core subunits (Ctf1 and Ssu72) and all three DPS complex subunits (Dis2, Ppn1, and Swd22) are dispensable for growth.

Whereas there have been great strides made in defining the architecture of the budding yeast and human 3’ processing machinery, including structures of individual subunits and subunit complexes [2–5], structural insights into the fission yeast CPF are relatively sparse. Vanoosthuyse et al. [1] showed by affinity purifications and mass spectrometry that deletion of Ppn1 precluded association of Swd22 and Dis2 with the CPF core, and of Swd22 and Dis2 with each other, but did not affect the integrity of the core itself. A Ppn1-Dis2 binary complex was formed in the absence of Swd22 but did not associate with the CPF core. The association of a Ppn1-Swd22 binary complex with the core was unaffected by deletion of Dis2. Their findings suggest that Ppn1 serves as a scaffold for binding Swd22 and Dis2 (presumably at independent sites on Ppn1) and that both Ppn1 and Swd22 (but not Dis2) are needed for association with the CPF core.

A much richer picture is emerging of the genetic interactions of the inessential fission yeast CPF subunits. There is considerable genetic redundancy within the CPF itself, whereby the effects of ablating one inessential subunit are buffered by other inessential CPF components, such that inactivation of both elicits synthetic lethality. For example: (i) *ppn1*Δ and *swd22*Δ are synthetically lethal with *ctf1*Δ; and (ii) a phosphatase-dead *ssu72-C13S* allele is synthetically lethal with *ppn1*Δ, *swd22*Δ, and *dis2*Δ [6]. Thus, there is functional cross-talk between the core and DPS.

Moreover, Ssu72, Ctf1, Dis2, Swd22, and Ppn1 are constituents of a recently identified genetic interactome connecting 3’ processing/termination, the Pol2 “CTD code,” inositol pyrophosphate (IPP) signaling, and the transcriptional control of fission yeast phosphate homeostasis [6,7]. The carboxyl-terminal domain (CTD) of the Rpb1 subunit of fission yeast Pol2 consists of tandemly repeated heptapeptides of consensus sequence Y^1^S^2^P^3^T^4^S^5^P^6^S^7^. The inherently plastic CTD structure is tuned by dynamic phosphorylation and dephosphorylation of the heptad serine, threonine, and tyrosine residues and by isomerization of the prolines between *trans* and *cis* configurations. These variations in the primary structure comprise a CTD code that conveys informational cues about the transcription machinery that are read by CTD-binding proteins [8–10]. Ser5 is the only strictly essential phosphorylation site in fission yeast. *S*. *pombe* is viable when the other phospho-sites are replaced by a non-phosphorylatable side chain (alanine for Ser2, Thr4, or Ser7; Phe for Tyr1) [6,11,12]. The findings that *ppn1*Δ and *swd22*Δ are synthetically lethal with *rpb1-CTD* mutants *Y1F*, *S2A*, and *T4A* suggested that Tyr1-Ser2-Thr4 form a three-letter CTD code “word” that abets 3’ processing/termination [6]. It is proposed that this CTD word is read by termination factor Rhn1, which recognizes the Thr4-PO_4_ CTD mark (6). Consistent with this idea, ChIP-seq analysis has shown that the Thr4-PO_4_ CTD mark is inscribed as Pol2 reaches the poly(A) site (PAS) of protein-coding genes and peaks shortly after the PAS [13]. The *rhn1*Δ null allele is synthetically lethal with *ppn1*Δ and *swd22*Δ, as are missense mutations of Rhn1 that perturb its CTD-binding site (6). *ppn1*Δ and *swd22*Δ are also synthetically lethal with *pin1*Δ, a null mutation of the Pin1 peptidyl-prolyl *cis-trans* isomerase that modifies the CTD [14]. The intersection of this genetic axis with IPP metabolism and phosphate homeostasis is described under Results.

In the present study, we focus on the DPS complex and its Ppn1 subunit. Ppn1 is a 710-amino acid polypeptide that is judged to be a homolog of mammalian PNUTS (PP1 NUclear Targeting Subunit), a regulator of the mammalian PP1 phosphoprotein phosphatase to which fission yeast Dis2 is homologous [1,15,16]. We take advantage of the several genetic backgrounds in which Ppn1 is essential for fission yeast growth (Fig 1) to conduct a structure-function analysis of Ppn1 aimed at: (i) defining which parts of the Ppn1 protein are dispensable and essential for Ppn1 activity in vivo; and (ii) assessing whether different structural features of Ppn1 come into play in different genetic contexts. We employ yeast two-hybrid assays to delineate distinct binding sites within Ppn1 for Dis2 and Swd22 and then gauge the contributions of these sites to Ppn1’s essential activities. In addition, we report RNA-seq analyses that identify sets of protein-coding genes that are concordantly up-regulated or down-regulated in *ppn1*Δ, *swd22*Δ, and *dis2*Δ cells.

## Results

### Transcription profiling of DPS null mutants

Ppn1, Swd22, and Dis2 comprise a discrete physical entity (the DPS module) within the fission yeast holo-CPF assembly [1]. Although DPS is inessential for vegetative growth, genetic analyses indicate that DPS deficiency is buffered by other factors that promote or affect 3’ processing and transcription termination, one of which is the CPF core subunit Ssu72 [6]. Recent RNA-seq studies show that inactivation of the Ssu72 protein phosphatase results in down-regulation or up-regulation of a narrow subset of fission yeast protein-coding genes [7]. By extension, we envision that: (i) the DPS complex also influences the expression of specific genes; and (ii) gauging the extent to which the effects of ablating individual DPS subunits on the transcriptome overlap and echo those of mutations in other transcription components might afford new insights to gene regulation. To address these issues, we performed RNA-seq on poly(A)^+^ RNA isolated from wild-type, *ppn1*Δ, *swd22*Δ, and *dis2*Δ cells. cDNAs obtained from three biological replicates (using RNA from cells grown to mid-log phase in YES medium at 30°C) were sequenced for each strain. In the datasets, 93–95% of the reads were mapped to unique genomic loci (S1 Fig). Read densities (RPKM) for individual genes were highly reproducible between biological replicates (Pearson coefficients of >0.98; S2 Fig). As internal controls, we affirmed that there were no reads for the deleted *ppn1*, *swd22*, or *dis2* coding sequence in the respective null strains. A cutoff of ±2-fold change in normalized transcript read level and a corrected *p*-value of ≤0.05 were the criteria applied to derive an initial list of differentially expressed annotated loci in the DPS mutants versus the wild-type control. We then focused on differentially expressed genes with average normalized read counts ≥100 in either the wild-type or null strains in order to eliminate transcripts that were expressed at very low levels in vegetative cells.

**Fig 2:**
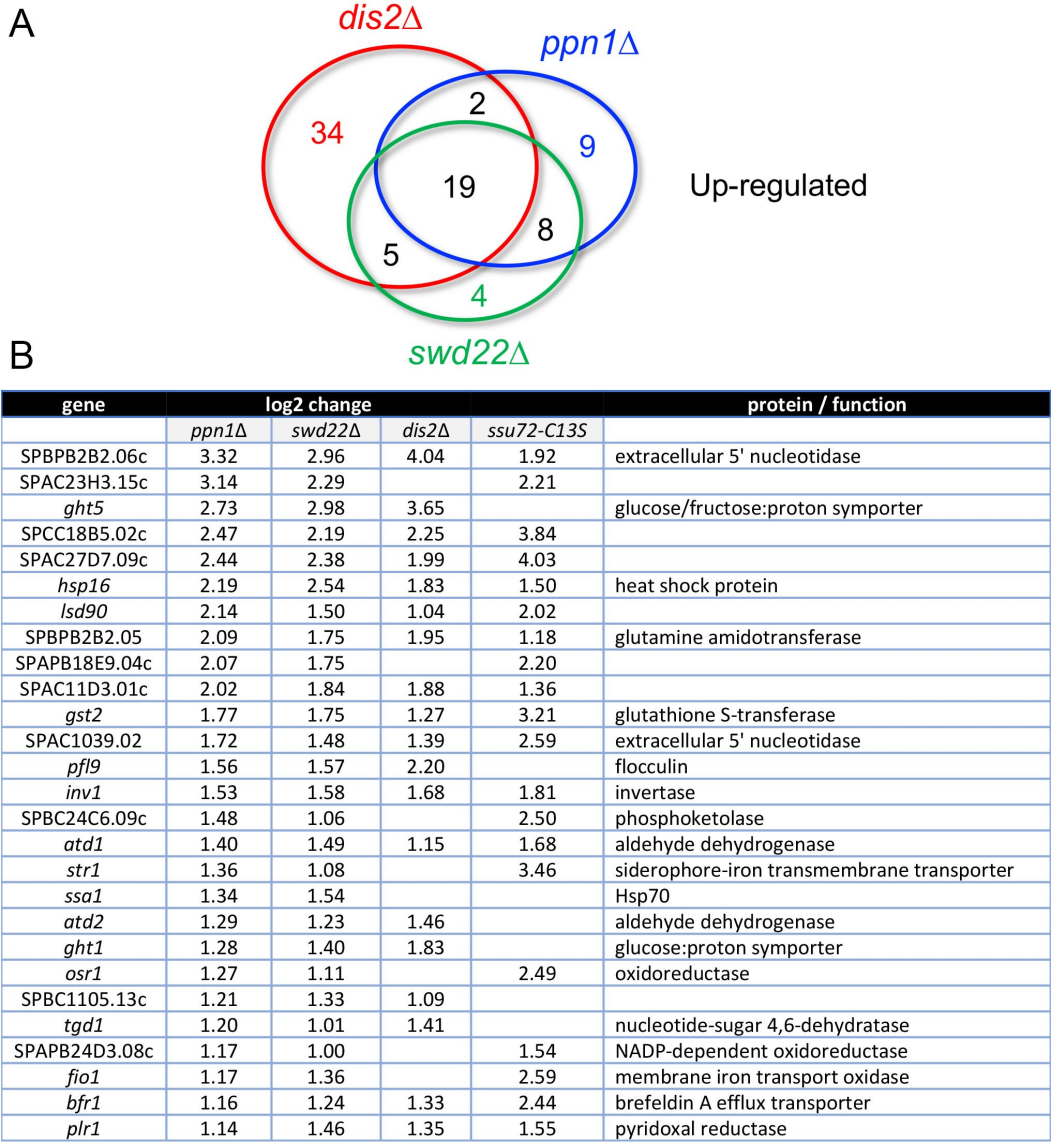
Coding genes up-regulated in DPS mutants. (A) Venn diagram depicting numbers of overlapping and non-overlapping up-regulated protein-coding genes. (B) List of 27 protein-coding genes that were coordinately up-regulated at least 2-fold in *ppn1*Δ and *swd22*Δ cells, 19 of which were also up-regulated in *dis2*Δ cells and 20 of which were up-regulated in s*su72-C13S* cells. The log2 fold changes versus wild-type are shown.

We thereby identified 38, 36, and 60 annotated protein-coding genes that were up-regulated in *ppn1*Δ, *swd22*Δ, and *dis2*Δ cells, respectively. The numbers of overlaps and uniquely affected transcripts are depicted in the Venn diagram in Fig 2A. The set of 27 coding genes that were coordinately up-regulated by ≥2-fold in the *ppn1*Δ and *swd22*Δ mutants is shown in Fig 2B (*p*-value <1.23e-55), of which 19 genes were also up-regulated in the *dis2*Δ strain. There was extensive overlap with the transcriptome of *ssu72-C13S* cells (7), whereby 20 coding genes up-regulated by *ssu72-C13S* were up-regulated by *ppn1*Δ and *swd22*Δ, 13 of which were also upregulated by *dis2*Δ (Fig 2B). The coordinately up-regulated gene sets included two involved in iron homeostasis, two 5’ nucleotidases, two heat-shock chaperones, and several oxidoreductases. Reference to the annotation of these transcription units in Pombase provided no immediate insights to their shared response to CPF mutations.

**Fig 3:**
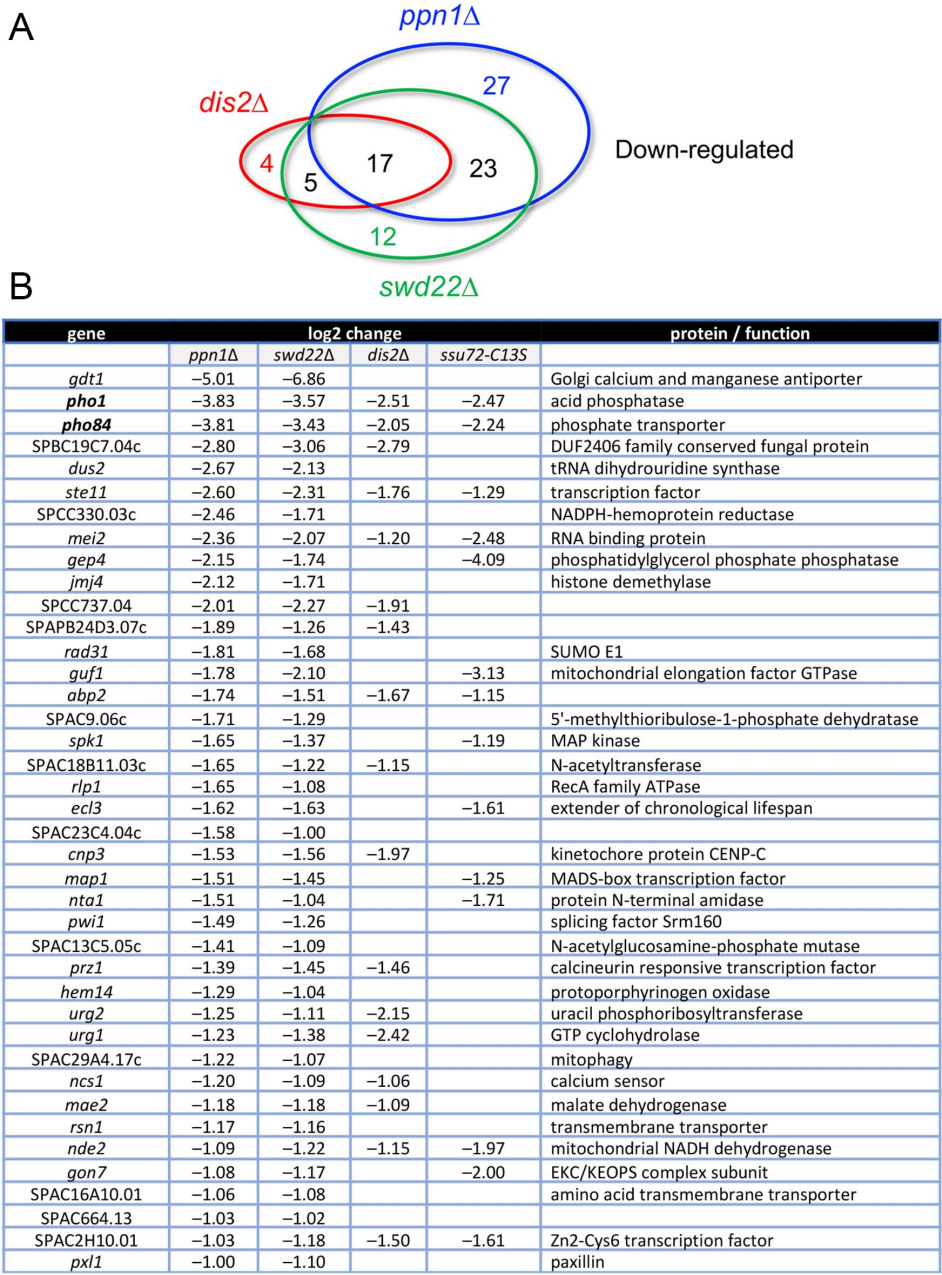
Coding genes down-regulated in DPS mutants. (A) Venn diagram depicting numbers of overlapping and non-overlapping down-regulated protein-coding genes. (B) List of 40 protein-coding genes that were coordinately down-regulated at least 2-fold in *ppn1*Δ and *swd22*Δ cells, 17 of which were also down-regulated in *dis2*Δ cells and 14 of which were down-regulated in s*su72-C13S* cells. The log2 fold changes versus wild-type are shown.

Our RNA-seq pinpointed 67, 57, and 26 protein-coding genes that were down-regulated by ≥2-fold in *ppn1*Δ, *swd22*Δ, and *dis2*Δ cells, respectively (Fig 3A). 40 coding transcripts were coordinately down-regulated in the *ppn1*Δ and *swd22*Δ mutants (*p* <3.29e-68), of which 17 were also down-regulated in *dis2*Δ cells and 14 were down-regulated in *ssu72-C13S* cells (Fig 3B). Among the most highly down-regulated transcripts in *DPS*Δ cells were those of two phosphate homeostasis genes–*pho1* and *pho84 –*that are under repressive control imposed by transcription of upstream flanking lncRNAs [17]. Previous studies had established that expression of the cell surface associated Pho1 acid phosphatase enzyme is hyper-repressed in *ppn1*Δ, *swd22*Δ, *dis2*Δ, and *ssu72-C13S* cells [6], an effect attributed to reduced efficiency of lncRNA 3’ processing and transcription termination prior to encounter of Pol2 with the *pho1* mRNA promoter, thereby enhancing lncRNA interference with mRNA expression. The Pombase annotation of the SPBC19C7.04c gene, which was downregulated ~8-fold in DPS mutants, suggests that it, too, might be subject to upstream transcription interference. SPBC19C7.04c encodes a 124-amino acid conserved fungal protein. The SPBC19C7.04c mRNA is annotated in PomBase as a 1450-nucleotide transcript that includes a 634-nucleotide 5’-UTR. The 5’-flanking gene, SPNCRNA.1553, specifies a 2044-nucleotide lncRNA that overlaps the putative 5’-UTR of the SPBC19C7.04c mRNA.

### Ppn1 truncation mutants

**Fig 4:**
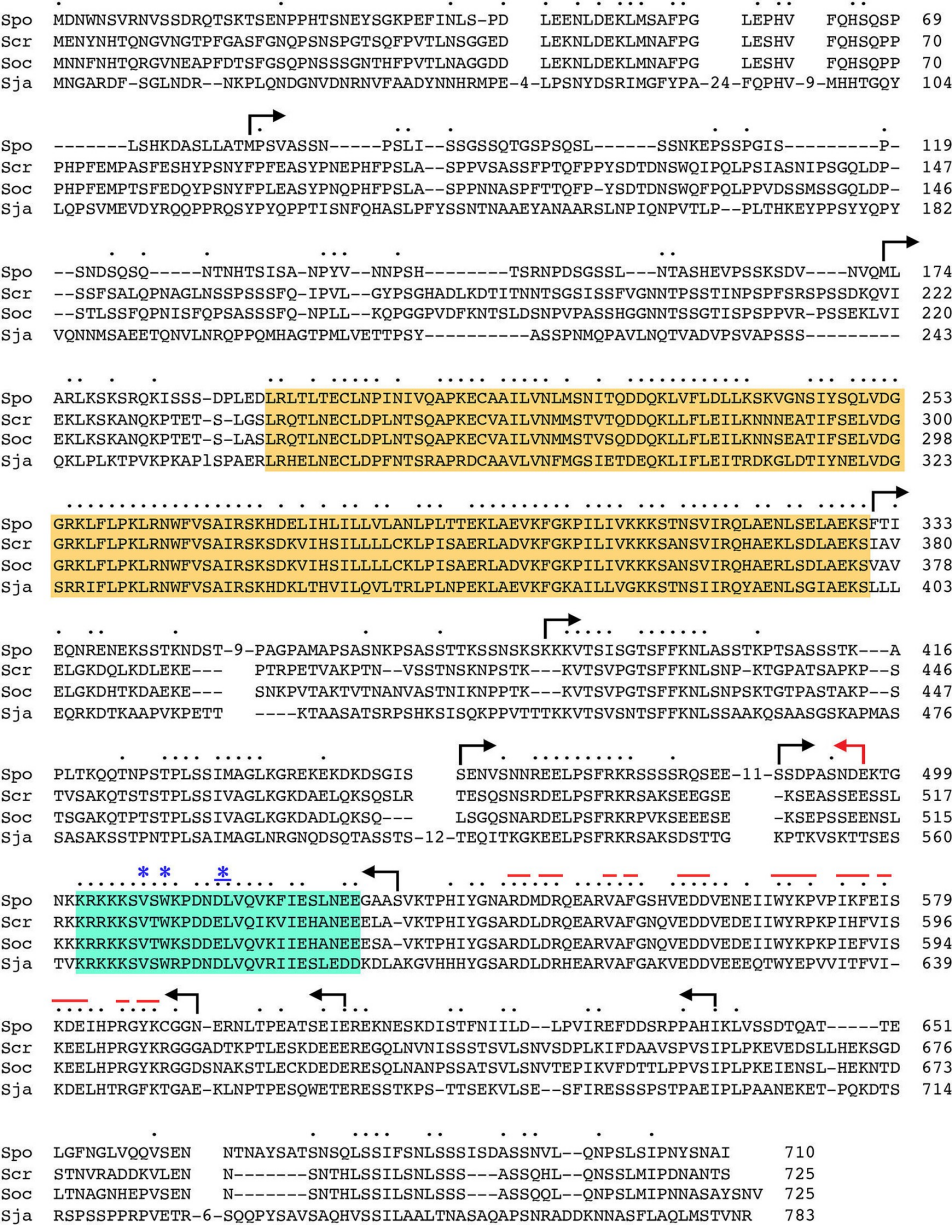
Primary structure of fission yeast Ppn1. The amino acid sequence of *S*. *pombe* (Spo) Ppn1 is aligned to those of the Ppn1 proteins from *S*. *cryophilus* (Scr), *S*. *octosporus* (Soc), and *S*. *japonicus* (Sja). Short gaps in the alignment are indicated by dashes; longer gaps are denoted by numbers of intervening amino acids. Positions of side chain identity/similarity in all four Ppn1 proteins are indicated by dots above the *S*. *pombe* sequence. Margins of serial N- and C-terminal truncations of *S*. *pombe* Ppn1 are shown as forward and reverse arrows. A conserved domain with predicted homology to the N-terminal TFIIS-like domain of rat PNUTS is highlighted in gold shading. A conserved segment that embraces the Dis2 binding site is highlighted in cyan shading; amino acids required for Ppn1-Dis2 interaction in a yeast 2-hybrid assay are denoted by asterisks above the alignment. The red lines above the alignment indicate residues within the Swd22 binding module of Ppn1 that were subjected to alanine scanning and testing of mutational effects on 2-hybrid protein-protein interactions.

**Fig 5:**
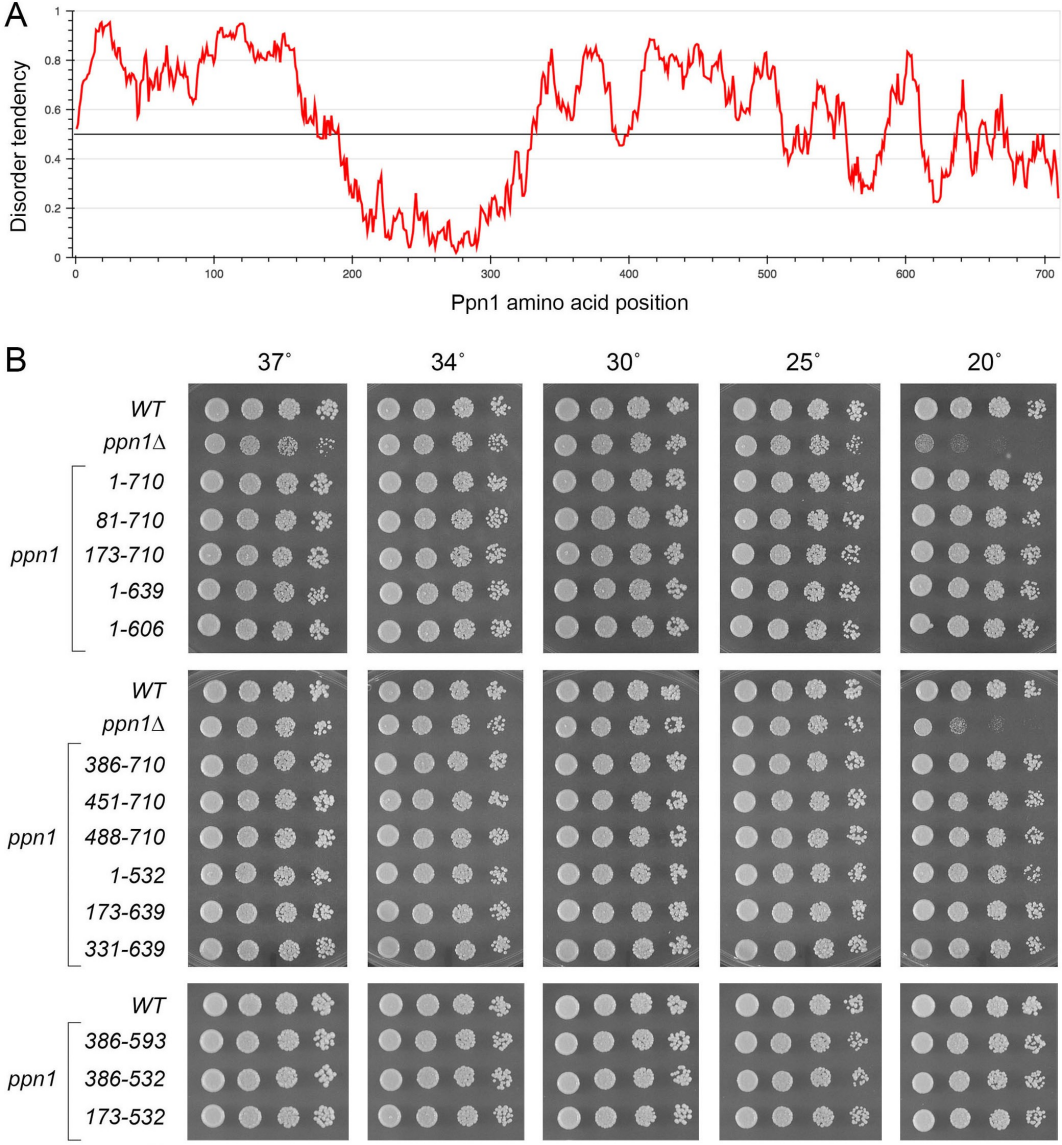
Fission yeast strains with *ppn1* truncation alleles. (A) An IUPred2A plot of disorder tendency by position in the *S*. *pombe* Ppn1 primary structure. (B) Serial dilutions of cultures of wild-type (*WT*), *ppn1*Δ, and the indicated *ppn1* truncation mutants were spot-tested for growth on YES agar at the temperatures specified.

We constructed a series of N-terminal and C-terminal deletion variants of *S*. *pombe* Ppn1, guided by an alignment of its primary structure to those of Ppn1 homologs from three other *Schizosaccharomyces* species (Fig 4) and a IUPred2A plot [18] of its disorder tendency by position in the primary structure (Fig 5A). The N-terminal 172-aa of *S*. *pombe* Ppn1 displays relatively scant conservation with its *S*. *cryophilus*, *S*. *octosporus*, and *S*. *japonicus* counterparts (33 positions of side chain identity/similarity in all four proteins) (Fig 4). This poorly conserved N-terminus is predicted to be disordered in its entirety (Fig 5A). The ensuing region from aa 194–330 (Fig 4, shaded in gold) is highly conserved among the four fission yeast Ppn1 proteins, with 102 positions of identity/similarity, and is predicted to be structurally ordered (Fig 5A). Analysis of this segment of *S*. *pombe* Ppn1 in Phyre2 [19] engendered a high-confidence (99.4%) structural homology model of Ppn1-(186–325) templated on the solution NMR structure of the N-terminal transcription elongation factor TFIIS-like domain of rat PNUTS [20]. The predicted all-helical fold of Ppn1-(186–325) is shown in stereo in S3 Fig. Alignment of the primary structures of the Ppn1 and PNUTS TFIIS-like modules reveals 48 positions of amino acid side chain identity/similarity (S3 Fig). The rest of the Ppn1 protein consists of local segments of high sequence conservation with other fission yeast Ppn1 homologs (aa 388–401, 427–436, 458–467, 502–528, and 533–589), some of which are predicted to have modest structural order (e.g., aa 511–520, 557–582), interspersed with poorly conserved segments that are predicted to be mostly disordered.

**Fig 6:**
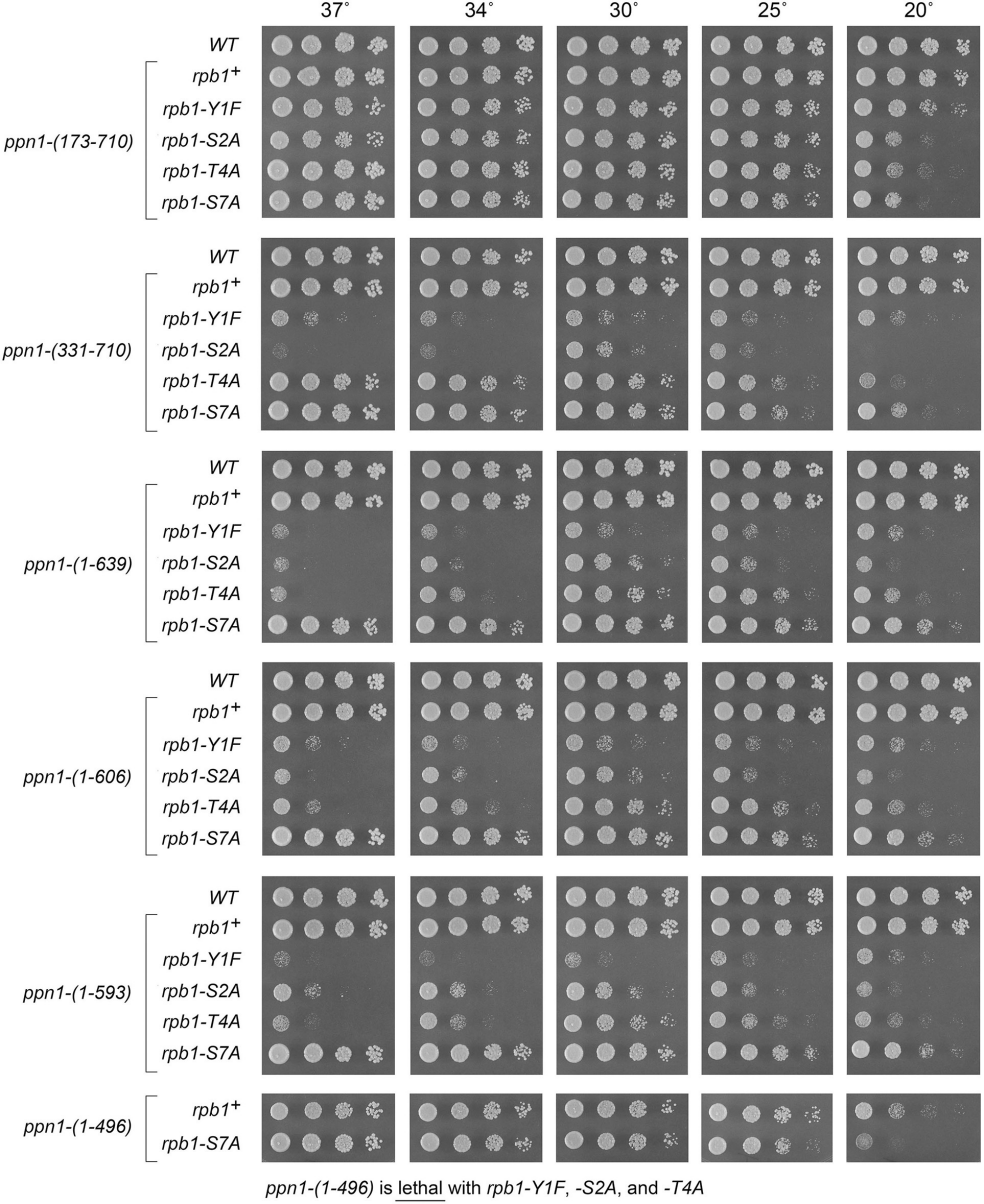
Activity of Ppn1 truncations in CTD mutant backgrounds in which Ppn1 is essential. *S*. *pombe* strains bearing the indicated *ppn1* truncation alleles in combination with *rpb1-CTD* phospho-site mutants were spot-tested for growth on YES agar at the indicated temperatures. The *ppn1-(1–496)* allele, which did not complement the *cs* growth phenotype seen in *ppn1*Δ, was lethal in the *rpb1-Y1F*, -*S2A*, and -*T4A* backgrounds.

We serially deleted 80, 172, 330, 385, 450, or 487 amino acids from the N-terminus or 71, 104, 117, 178, or 214 amino acids from the C-terminus of *S*. *pombe* Ppn1 (the margins of the truncations are indicated by arrows in Fig 4). Full-length and truncated *ppn1* alleles, marked by a 3’-flanking drug-resistance cassette, were inserted into the chromosomal *ppn1* locus in lieu of the native *ppn1*^+^ gene and the resulting strains were spot tested for growth on YES agar at 20, 25, 30, 34, and 37°C in parallel with: (i) an unmarked wild-type *ppn1*^+^ strain (*WT*) that grows well at all temperatures and (ii) a *ppn1*Δ null mutant, which displays a cold-sensitive (*cs*) growth defect at 20°C (Fig 5B). All of the N-terminal deletion alleles, up to and including *ppn1-(488–710)*, complemented the *cs* defect, as did the C-terminal deletion alleles, up to and including *ppn1-(1–532)* (Fig 5B). However, a further C-terminal truncation allele *ppn1-(1–496)* was unable to sustain normal growth at 20°C (Fig 6). These results indicate that: (i) the N-terminal 487 amino acids (that includes the disordered N-terminus and the adjacent TFIIS-like domain) and the C-terminal 178 amino acids are individually dispensable for growth at cold temperatures; and (ii) the segment from aa 488–532, which includes a region of strong conservation among Ppn1 homologs (highlighted in cyan in Fig 4) is necessary for growth in the cold. We proceeded to test simultaneous deletions at both ends of Ppn1 and found that alleles *ppn1-(173–639)*, *ppn1-(331–639)*, *ppn1-(386–593)*, *ppn1-(173–532)*, and *ppn1-(386–532)*, supported growth at 20°C (Fig 5B).

To gauge the effects of Ppn1 truncations on the intracellular levels of Ppn1 protein, we raised rabbit polyclonal antibodies against purified recombinant Ppn1-(173–496) produced in *E*. *coli*. Whole cell extracts of fission yeast strains (normalized for total protein content) were resolved by SDS-PAGE, transferred to membrane, and subjected to Western blotting with affinity-purified anti-Ppn1 antibody. A ~90 kDa immunoreactive polypeptide corresponding to full-length Ppn1 (aa 1–710) was present in the wild-type strain but absent in *ppn1*Δ cells (S4 Fig). Incrementally smaller immunoreactive Ppn1 proteins of appropriate size were seen in cells expressing N-terminal truncations Ppn1-(173–710), -(331–710) and -(386–710) and bilateral truncations Ppn1-(173–639) -(331–639), and -(173–532) (S4 Fig). A noteworthy finding was that the steady-state level of Ppn1-(173–710) was clearly elevated compared to that of full-length Ppn1, as was the steady state level of the other five aforementioned truncated Ppn1 polypeptides (S4 Fig). These findings suggest that the disordered N-terminal 172-aa segment of Ppn1 has a destabilizing influence on Ppn1. Whereas acute decrements in the level of immunoreactive Ppn1 polypeptide were seen when the N-terminal 385-aa deletion was combined with C-terminal truncations at positions 593 and 532, respectively, the steady state levels of the Ppn1-(386–532) and Ppn1-(386–593) proteins were still similar to, for (386–532), or slightly higher than, for (386–593), that of full-length Ppn1 (S4 Fig). Immunoreactive C-terminally truncated Ppn1 proteins of incrementally smaller size were detected in *ppn1-(1–639)*, *ppn1-(1–593*), *ppn1-(1–532)*, and *ppn1-(1–496)* cells (S5 Fig).

### Activity of Ppn1 truncations in CTD mutant backgrounds in which Ppn1 is essential

**Fig 7:**
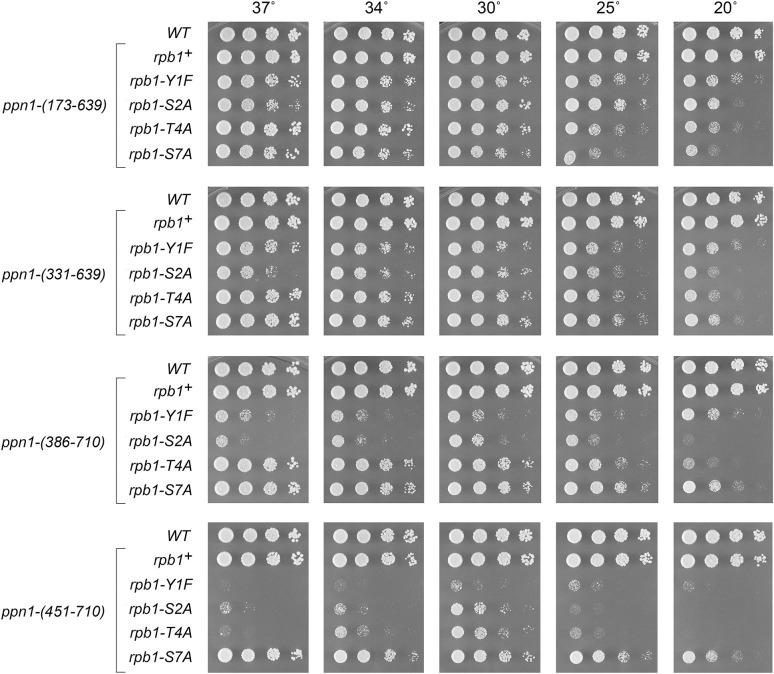
Activity of Ppn1 truncations in CTD mutant backgrounds. Strains with the indicated *ppn1* truncation alleles in combination with *rpb1-CTD* phospho-site mutants were spot-tested for growth on YES agar at the indicated temperatures.

*S*. *pombe rpb1-CTD* mutant strains, in which the native CTD length was maintained and Tyr1, Ser2, Thr4, or Ser7 in every consensus heptad was replaced by Phe, Ala, Ala, or Ala, respectively, thrive on YES agar medium at 30°C but grow slowly at 20°C [6]. *ppn1*Δ is synthetically lethal in the *CTD-Y1F*, *-S2A*, and *-T4A* backgrounds, but viable in combination with *CTD-S7A* [6]. Here we tested the capacity of the various *ppn1* truncation mutants to support viability in the three mutant *CTD* genetic backgrounds in which Ppn1 is essential. Haploid strains with *rpb1-CTD* alleles *Y1F*, *S2A*, *T4A*, and *S7A* (marked with a 3’ flanking *natMX* gene) were mated to haploid strains with *ppn1* truncation mutations. The resulting heterozygous diploids were sporulated and, for each allelic pair, a random collection of 500 to 1000 viable haploid progeny were screened by serial replica-plating for the presence of the flanking markers. A failure to recover any viable haploids with both markers, while recovering the three other haploid progeny (unmarked and the two singly marked haploids) with the expected frequencies, was taken as evidence of synthetic lethality between the CTD allele and the test *ppn1* allele. The double-mutant haploids that passed selection were spotted on YES agar at 20 to 37°C in parallel with two control strains, in which *rpb1* and *ppn1* were both wild-type or *rpb1* was wild-type and *ppn1* was truncated (Figs 6 and 7). As expected, all *ppn1* truncations were viable in combination with *CTD-S7A*.

We found that whereas N-terminal truncation *ppn1-(173–710)* was fully active in supporting growth of the *Y1F*, *S2A*, and *T4A* strains, *ppn1-(331–710)* caused a severe growth defect in *Y1F* and *S2A* cells (reflected in tiny colony size) but relatively little impact in the *T4A* background (Fig 6). *ppn1-(386–710)* phenocopied *ppn1-(331–710)* (Fig 7). *ppn1-(451–710)* was barely viable in combination with *Y1F* and was severely sick with *S2A* and *T4A* (Fig 7). C-terminal deletion *ppn1-(1–639)* resulted in failure of *Y1F*, *S2A*, and *T4A* cells to thrive at 37°C as well as synthetic sickness at 30°C in the *Y1F* and *S2A* backgrounds. Serial C-terminal deletions *ppn1-(1–606)* and *ppn1-(1–593)* phenocopied *ppn1-(1–639)* in the *CTD-Ala* strains, but further truncation to *ppn1-(1–496)* was synthetically lethal with *Y1F*, *S2A*, and *T4A* (Fig 6). We surmise from these results that: (i) the N-terminal 172-aa segment of Ppn1 is inessential; (ii) deletion of aa 173–330 or 640–710 elicits hypomorphic phenotypes in CTD mutant backgrounds, to which *Y1F* and *S2A* are more sensitive than *T4A*; and (iii) the segment from aa 452–593 contains elements necessary for Ppn1 function in CTD mutant backgrounds.

Testing combinations of N- and C-terminal truncations generated additional instructive findings. For example, *ppn1-(173–639)* and *ppn1-(331–639)* supported better growth of *Y1F*, *S2A*, and *T4A* cells, especially at 34°C and 37°C, than did *ppn1-(1–639)* (Fig 7, compare to Fig 6), suggesting that the disordered N-terminus exerts an inhibitory effect on Ppn1 function when the C-terminal 71-aa are missing. The growth phenotypes of *ppn1* truncation alleles in combination with CTD mutants are summarized in S6 Fig.

### Activity of Ppn1 truncations in 3’ processing/termination mutant backgrounds

**Fig 8:**
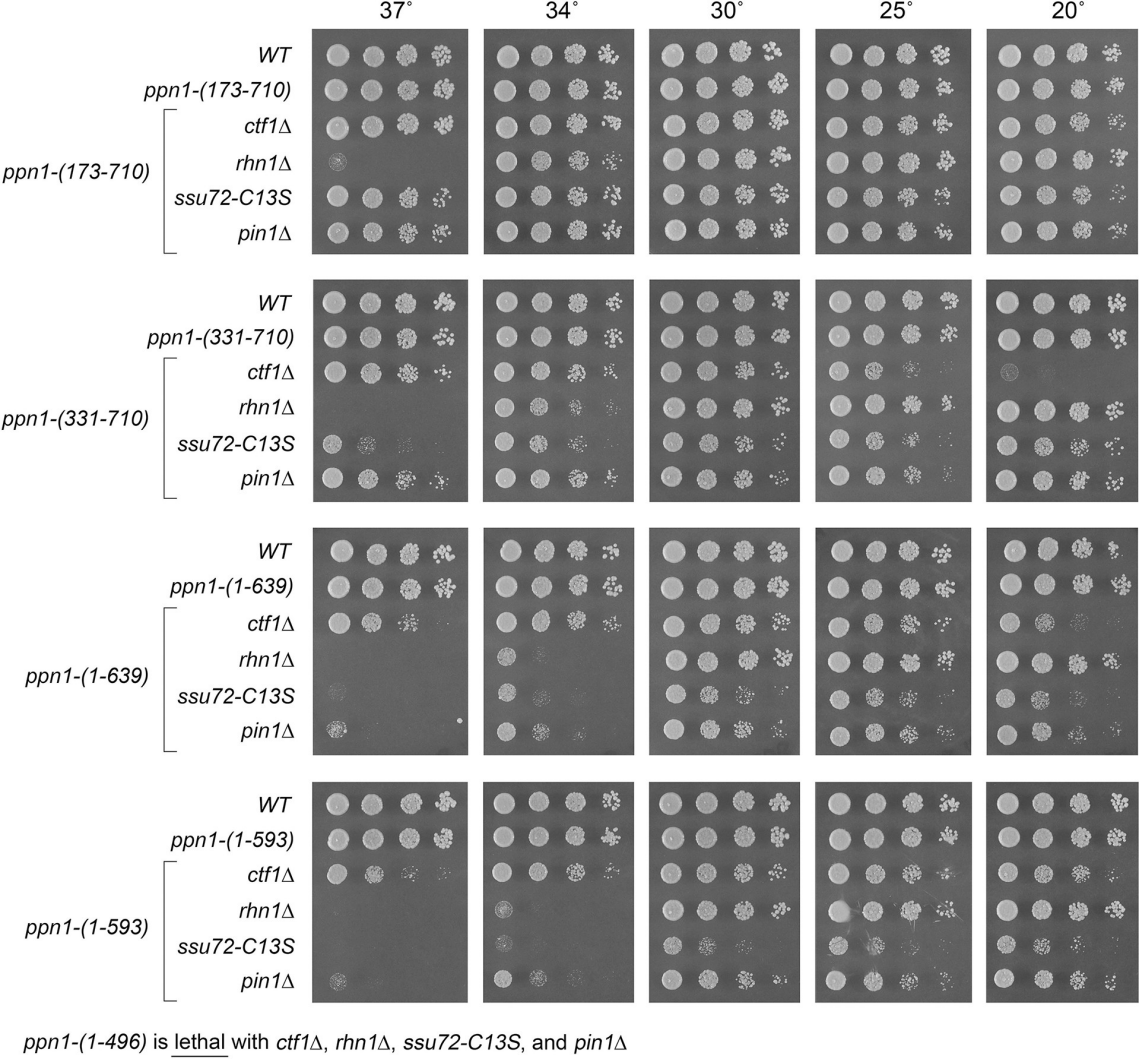
Activity of Ppn1 truncations in 3’ processing/termination mutant backgrounds. *S*. *pombe* strains bearing the indicated *ppn1* truncation alleles in combination with CPF subunit, Rhn1, or Pin1 mutants as specified were spot-tested for growth on YES agar at the indicated temperatures. The *ppn1-(1–496)* allele was synthetically lethal with *ctf1*Δ, *rhn1*Δ, *ssu72-C13S*, and *pin1*Δ.

**Fig 9:**
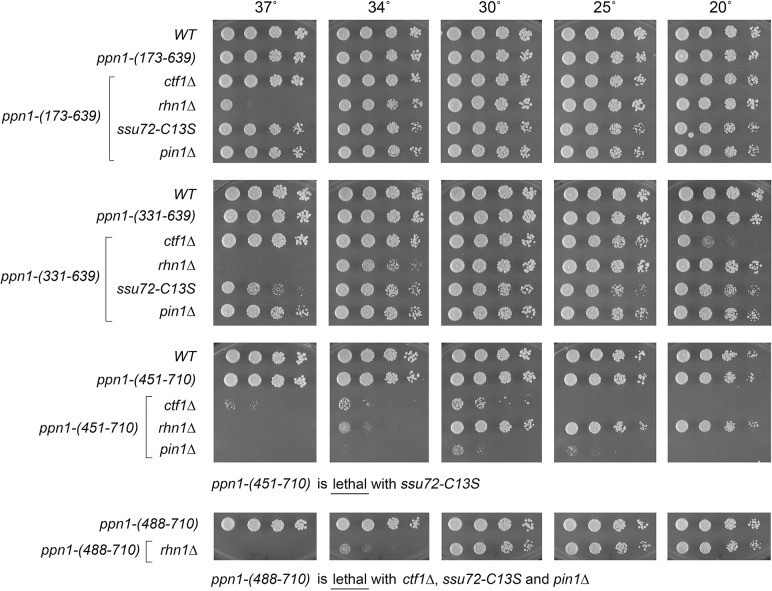
Activity of Ppn1 truncations in CPF/Rhn1/Pin1 mutant backgrounds. Strains with the indicated *ppn1* truncation alleles in combination with CPF subunit, Rhn1, or Pin1 mutants as specified were spot-tested for growth on YES agar at the indicated temperatures. Synthetic lethalities of *ppn1-(451–710)* and *ppn1-(488–710)* are specified below the panels.

Ppn1 is essential for growth in the absence of CPF core subunit Ctf1, when the *cis*-proline-requiring Ssu72 CTD phosphatase subunit of CPF core is either deleted or crippled by an active site mutation C13S, and in the absence of the CTD-binding transcription termination factor Rhn1 [6]. Ppn1 is also essential in the absence of Pin1, a peptidyl prolyl isomerase that promotes 3’ processing/termination via Ssu72 [14]. Here the *ppn1* truncation alleles were tested for activity in sustaining growth of *ctf1*Δ, *ssu72-C13S*, *rhn1*Δ, and *pin1*Δ cells (via mating and sporulation as described above for the CTD mutants). The results are shown in Figs 8 and 9. (Note that *rhn1*Δ single-mutant cells fail to grow at 37°C.) Key findings are as follows. N-terminal truncation *ppn1-(173–710)* was fully active in supporting growth of the four processing/termination mutants. *ppn1-(331–710)* was *cs* in the *ctf1*Δ strain and *ts* in the *ssu72-C13S* strain (Fig 8). *ppn1-(451–710)* exacerbated the growth phenotypes of *rhn1*Δ, *ctf1*Δ, and *pin1*Δ and was lethal in combination with *ssu72-C13S* (Fig 9). *ppn1-(488–710)* was lethal with *ctf1*Δ, *ssu72-C13S*, and *pin1*Δ but thrived in the *rhn1*Δ background at 20–30°C (Fig 9). C-terminal truncations *ppn1-(1–639)* and *ppn1-(1–593)* exacerbated the *ts* defect of *rhn1*Δ (seen as failure to thrive at 34°C) and elicited *ts* growth defects in *ssu72-C13S* and *pin1*Δ cells at 34 and 37°C (Fig 8). *ppn1-(1–496)* was uniformly lethal in combination with *ctf1*Δ, *ssu72-C13S*, *rhn1*Δ, or *pin1*Δ. The bilateral deletion alleles *ppn1-(173–639)* and *ppn1-(331–639)* grew better than *ppn1-(1–639)* in all four of the 3’ processing/termination mutant contexts (Fig 9). These results (summarized in S6 Fig) echo those obtained for the CTD mutants, insofar as the N-terminal disordered region of Ppn1 is dispensable in all genetic assays of Ppn1 essentiality, deleting the disordered N-terminus alleviates hypomorphic phenotypes caused by deleting the C-terminal segment from aa 640–710, and intermediate truncations have allele-specific severities, with *rhn1*Δ being less sensitive to hypomorphic mutations than *ctf1*Δ, *ssu72-C13S*, and *pin1*Δ.

### Effect of Ppn1 truncations on phosphate homeostasis

**Fig 10:**
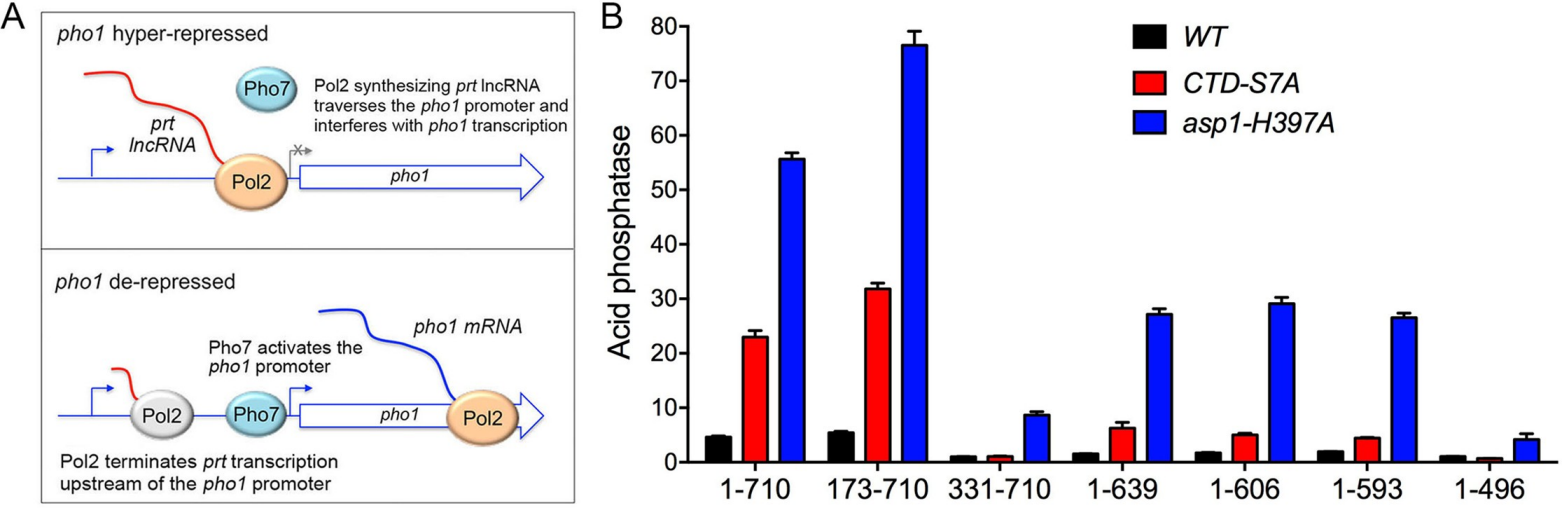
Effect of Ppn1 truncations on phosphate homeostasis. (A) Top panel. Decreased termination of *prt* lncRNA synthesis increases interference with the *pho1* mRNA promoter and hyper-represses Pho1 expression in phosphate-replete cells. Bottom panel. Precocious termination of *prt* lncRNA synthesis upstream of the *pho1* mRNA promoter results in de-repression of Pho1. See text for discussion. (B) *S*. *pombe* strains bearing the *ppn1* alleles shown on the x-axis, in an otherwise wild-type background (WT) or in the context of the *pho1* de-repressive *rpb1-CTD-S7A* or *asp1-H397A* mutations, were grown in liquid culture at 30°C and assayed for acid phosphatase activity.

Fission yeast phosphate homeostasis is a transcriptional program that is governed by the Pol2 CTD code and the 3’ processing/termination machinery [6,21,22]. The *S*. *pombe* phosphate regulon comprises three genes that specify, respectively, a cell surface acid phosphatase Pho1, an inorganic phosphate transporter Pho84, and a glycerophosphate transporter Tgp1 [23]. Expression of *pho1*, *pho84*, and *tgp1* is actively repressed during growth in phosphate-rich medium by the transcription in *cis* of a long noncoding (lnc) RNA from the respective 5’ flanking genes *prt*, *prt2*, and *nc-tgp1* [24–29]. A model for the repressive arm of fission yeast phosphate homeostasis is that transcription of the upstream lncRNA interferes with expression of the downstream mRNA genes by displacing the activating transcription factor Pho7 from its binding site(s) in the mRNA promoters that overlap the lncRNA transcription units [17]. A Pol2 *CTD-S7A* allele that prevents installation of the Ser7-PO_4_ mark de-represses *PHO* genes in phosphate-replete cells [6,21,22] by promoting precocious termination of upstream lncRNA transcription prior to encounter of Pol2 with the downstream mRNA promoter (Fig 10A). This model is supported by findings that: (i) mutations of CPF subunits (including *ppn1*Δ), Rhn1, and Pin1 –proteins that normally promote 3’ processing/termination–result in hyper-repression of *pho1* under phosphate-replete conditions; and (ii) the de-repression of *pho1* elicited by the *CTD-S7A* allele is erased by mutations of CPF subunits (including *ppn1*Δ), Rhn1, and Pin1 [6,14]. Recent studies also implicate the inositol pyrophosphate (IPP) signaling molecule IP8 as an agent in the nexus of the CTD code with 3’ processing/termination, based on biochemical phenotypes and mutational synergies elicited by genetic manipulations of Asp1 [7], a bifunctional enzyme composed of an N-terminal IPP kinase domain and a C-terminal IPP pyrophosphatase domain [30,31]. The function of the Asp1 kinase is to generate 1,5-IP8 via phosphorylation of its substrate 5-IP7 and the function of the Asp1 pyrophosphatase is to convert its substrate 1,5-IP8 back to 5-IP7. An IPP-pyrophosphatase-dead allele *asp1-H397A* that results in elevated intracellular levels of IP8 elicits a strong de-repression of the *PHO* regulon that correlates, in the case of the *prt–pho1* locus, with precocious termination of *prt* lncRNA transcription [7]. The de-repression of *pho1* by *asp1-H397A* is erased by mutations of CPF subunits (including *ppn1*Δ), Rhn1, and Pin1 [7,14].

Here we queried the effect of *ppn1* truncations on *pho1* expression during exponential growth at 30°C in liquid culture under phosphate-replete conditions, *per se*, and in the *CTD-S7A* and *asp1-H397A* genetic backgrounds in which *pho1* is de-repressed in a manner that relies on Ppn1 function (Fig 10B). Acid phosphatase activity, a gauge of Pho1 enzyme level, was quantified by incubating suspensions of serial dilutions of the phosphate-replete cells for 5 min with *p*-nitrophenyl phosphate and assaying colorimetrically the formation of *p*-nitrophenol. The basal Pho1 activity of wild-type cells was unaffected in *ppn1-(173–710)* cells [*vis-à-vis* the *ppn1-(1–710)* wild-type strain] but was hyper-repressed in *ppn1-(1–496)* cells expressing a non-functional Ppn1 mutant. The de-repression of Pho1 seen in *CTD-S7A* and *asp1-H397A* cells expressing wild-type Ppn1 was erased in the *ppn1-(1–496)* strain (Fig 10B), as shown previously for *ppn1*Δ [7]. A noteworthy finding was that the de-repressed levels of Pho1 activity in *ppn1-(173–710) CTD-S7A* and *ppn1-(173–710) asp1-H397A* cells were 38% higher than in the respective *ppn1-(1–710)* controls (Fig 10B). By contrast, the *ppn1-(331–710)* allele elicited Pho1 hyper-repression *per se* and eliminated (in *CTD-S7A* cells) or severely attenuated (in *asp1-H397A* cells) Pho1 de-repression (Fig 10B). We surmise that the segment from aa 173–330 that includes the predicted TFIIS-like module is important for Ppn1 function in *prt* lncRNA termination. The C-terminal truncation alleles *ppn1-(1–639*), *-(1–606)*, and *-(1–593)* were hyper-repressive *per se* and partially attenuated the de-repression of Pho1 by *CTD-S7A* and *asp1-H397A* (Fig 10B), consistent with our designation of these mutants as functional hypomorphs.

### Yeast 2-hybrid assays identify distinct Dis2 and Swd22 binding sites in Ppn1

**Fig 11:**
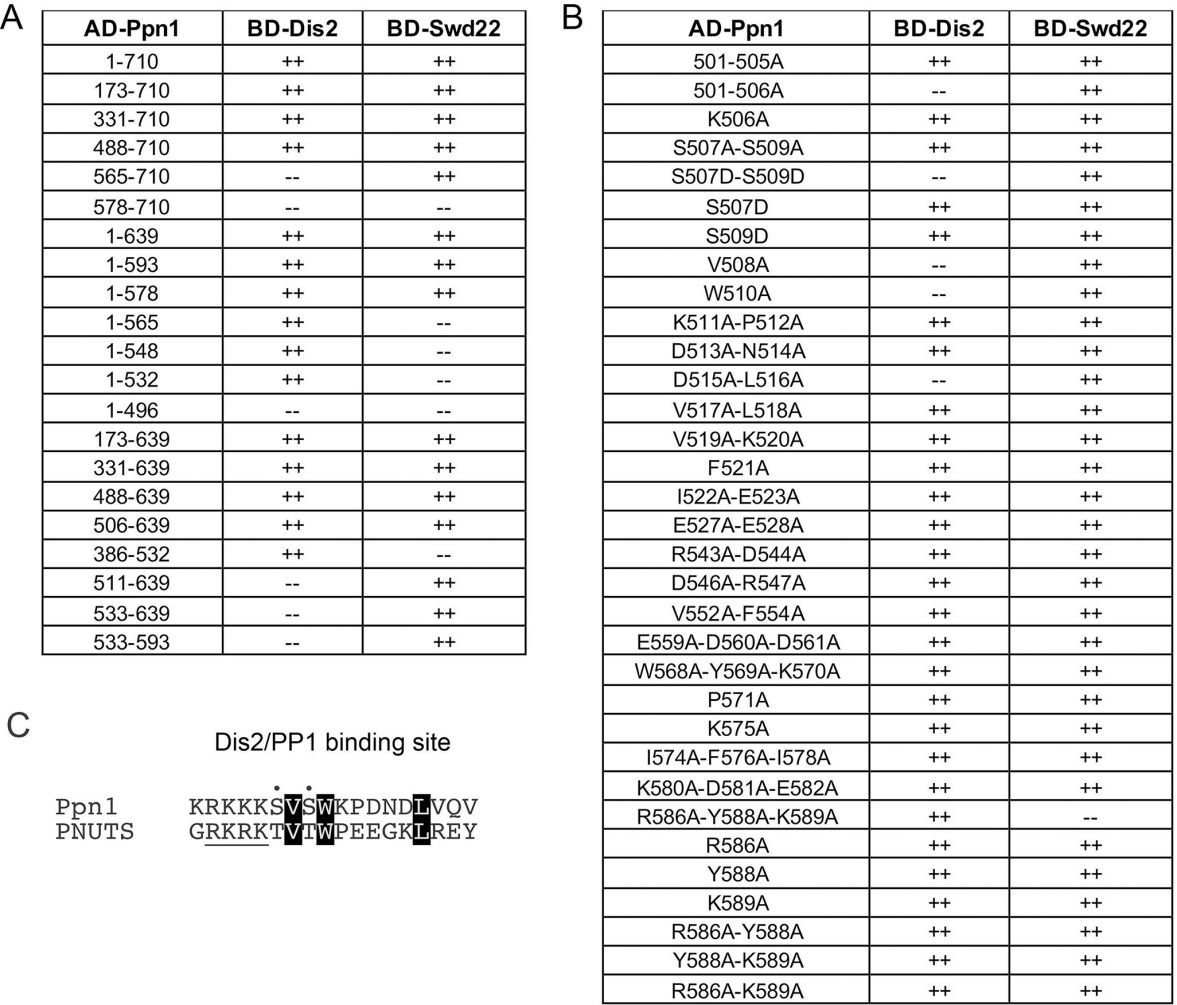
Yeast 2-hybrid assays identify distinct Dis2 and Swd22 binding sites in Ppn1. (A) Full-length Ppn1 (aa 1–710) and the indicated Ppn1 fragments fused to the Gal4 AD were tested for 2-hybrid interactions with Gal4 BD fusions to full-length Dis2 and full-length Swd22. AD/BD pairs that scored positive in both reporter assays (LacZ expression and histidine prototrophy) are indicated as ++. Pairs that were negative in both reporter assays are scored as --. (B) Full-length AD-Ppn1 constructs with the indicated amino acid substitutions were tested for 2-hybrid interactions with BD-Dis2 and BD-Swd22. (C) The amino acid sequences of the PP1/Dis2 binding motifs in PNUTS and Ppn1 are aligned. The conserved hydrophobic amino acids important for the Ppn1-Dis2 interaction in the 2-hybrid assay (Val, Trp, Leu) are in white font on black background. Conserved hydroxyamino acids that are potential phosphorylation sites are denoted by dots. The upstream basic patch is underlined.

Full-length and truncated versions of Ppn1 were tested for interaction with DPS subunits Dis2 and Swd22 in the yeast 2-hybrid assay, with dual reporter readouts (His^+^ and lacZ^+^) of a positive interaction between a transcription activation domain (AD) fused to Ppn1 and a DNA binding domain (BD) fused to full-length Dis2 or Swd22 (scored as ++ in Fig 11A). Full-length Ppn1 interacted with both Dis2 and Swd22, signifying that none of the subunits of the *S*. *pombe* CPF core are needed for binary interactions of Ppn1. Deleting up to 505 amino acids from the N-terminus did not affect Ppn1 interaction with Dis2 and Swd22. Both interactions were preserved when the C-terminus of Ppn1 was truncated at amino acid 593 (Fig 11A). By testing various combinations of N and C terminal truncations, we defined an internal segment from 506–639 as sufficient for Ppn1 interaction with Dis2 and Swd22 (Fig 11A). Neither the N-terminal segment from aa 1–496 nor the C-terminal fragment from 578–710 was able to bind to Dis2 or Swd22 in the 2-hybrid format (Fig 11A).

The findings that: (i) Ppn1-(1–565), -(1–548), -(1–532), and -(386–532) bound to Dis2 but not to Swd22; and (ii) Ppn1-(511–639), -(533–639), and -(533–593) bound to Swd22 but not Dis2 (Fig 11A) engender the following inferences. First, that Dis2 and Swd22 bind to distinct sites on Ppn1. Second, that the Dis2 binding site is localized within the Ppn1 segment from aa 506–532 and requires the peptide ^506^KSVSW^510^. Third, that the Swd22 binding site is located in the segment from aa 533–578 and requires the segment from aa 566–578.

### 2-hybrid alanine scan of the Dis2 and Swd22 binding regions of Ppn1

The 27-aa segment that encompasses the Dis2 binding site (shaded cyan in Fig 4) is highly conserved among the four *Schizosaccharomyces* Ppn1 homologs. In an effort to identify essential constituents of the Dis2 interface, we introduced single-alanine and double-alanine mutations into the full-length AD-Ppn1 2-hybrid bait construct, targeting every amino acid from Lys506 to Glu523, plus Glu527 and Glu528. Controls showed that none of the alanine mutations affected Ppn1 interaction with Swd22 (Fig 11B). Ppn1 binding to Dis2 was abolished by single alanine changes V508A and W510A and by the D515A-L516A double-mutant (Fig 11B). None of the other alanine substitutions of 16 amino acids in the Dis2 box (mostly double-alanine mutants) affected Dis2 binding in the 2-hybrid format. The strictly conserved Val508 and Trp510 residues essential for Dis2 binding are indicated by blue asterisks in Fig 4. The Asp515-Leu516 dipeptide needed for Dis2 interaction is either Asp-Leu or Glu-Leu in the three other *Schizosaccharomyces* Ppn1 homologs (Fig 4). We suggest that the ^508^VxW^510^ motif in Ppn1 is the putative counterpart of a Vx(F/W) motif that comprises a key part of the PP1 phosphatase-binding site identified in several other PP1 regulatory subunits, including PNUTS, wherein the Vx(F/W) motif binds to a hydrophobic pocket of the phosphatase remote from the phosphatase active site [32–35] (Figs 11C and S7). A leucine equivalent of Ppn1 Leu516 is a conserved constituent of the hydrophobic PP1-binding interface of other phosphatase regulators, including PNUTS (Fig 11C and S7).

The proximal edge of the Dis2-binding “box” in Ppn1 consists of a run of six basic amino acids (^501^KKRKKK^506^), five of which are conserved in all three other fission yeast Ppn1 homologs (Fig 4). Other well-studied PP1 phosphatase-regulatory proteins have at least one, and often several, basic amino acids preceding the Vx(F/W) motif [32–35]. In the case of PNUTS, there is a run of four basic amino acids upstream of VxW (Fig 11C); two of these basic amino acids engage in salt bridges to acidic side chain chains of PP1 (S7 Fig). Because mutating Lys506 singly to alanine did not affect Dis2 interaction with full-length Ppn1, we proceeded to change all six basic amino acids to alanine simultaneously. The 501-506A mutation of Ppn1 eliminated Dis2 interaction without affecting Swd22 interaction (Fig 11B). Yet changing only the first five of the basic amino acids to alanine did not affect the 2-hybrid interaction of Ppn1-(501-505A) with Dis2 (Fig 11B). Thus, at least one basic side chain in this basic patch is needed for Dis2 interaction.

The Ppn1 Dis2-binding box contains two serines, Ser507 and Ser509, that are conserved as serine or threonine in the three other fission yeast Ppn1 proteins (Fig 4) and in PNUTS (Fig 11C). Previous studies showed that interaction of PNUTS with PP1 phosphatase can be modulated negatively by threonine phosphorylation at the PP1-binding site (RKRKTVTW) of PNUTS [16]. To query whether serine phosphorylation might affect Ppn1 interaction with Dis2, we introduced phosphomimetic aspartate substitutions at Ser507 and Ser509 of full-length Ppn1, singly and in combination, and tested their effects in the 2-hybrid assay. The single S507D and S509D changes did not compromise Ppn1 interaction with Dis2, but the S507D-S509D double-mutation abolished Dis2 interaction without affecting Ppn1 binding to Swd22 (Fig 11B). Thus, the simultaneous acquisition of negative charge at these two sites was inimical to the Dis2 binding interface of Ppn1. Mutation of both serines to alanine did not disrupt Ppn1-Dis2 interaction.

To interrogate the Swd22 interface, we constructed 10 single-alanine, double-alanine and triple-alanine mutations in the full-length AD-Ppn1 2-hybrid expression vector, targeting 23 amino acids denoted by the red lines in Fig 4. Nine of the Ppn1-Ala mutants did not affect the 2-hybrid interaction with Swd22 (or with the Dis2 control). Only the R586A-Y588A-K589A triple-mutant failed to score as interacting with Swd22, while maintaining interaction with Dis2 (Fig 11B). To deconvolute the Swd22 binding-defective triple-mutant, we changed each amino acid singly to alanine and made all three combinations of double-alanine substitutions, none of which compromised the 2-hybrid interaction with Swd22 (Fig 11B).

### Effect of Dis2 binding-defective mutations on Ppn1 activity in vivo

**Fig 12:**
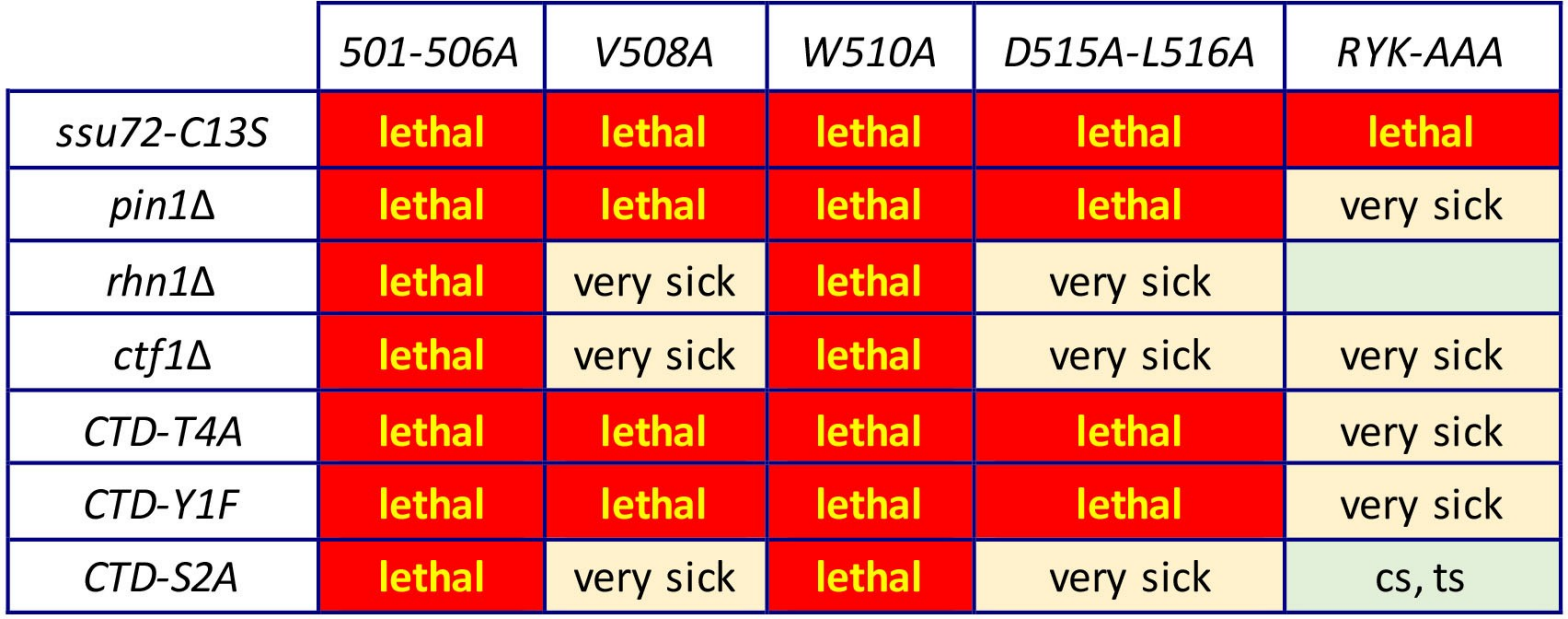
Effect of Dis2 and Swd22 binding-defective mutations on Ppn1 activity in vivo. *ppn1* mutants specified in the top row were crossed to the mutant strains specified in the left-most column. Synthetically lethal pairs of alleles are highlighted in red boxes. The yellow boxes indicate severe synthetic growth defects (very sick). A viable double mutant without a synthetic defect is indicated by a plain green box. A viable double mutant that displayed temperature-sensitive (*ts*) or cold-sensitive (*cs*) defects is annotated as such in its green box.

The *501-506A*, *V508A*, *W510A*, and *D515A-L516A* mutations that selectively interdicted Ppn1 binding to Dis2 in the 2-hybrid assay were introduced into the fission yeast chromosomal *ppn1* locus. The Dis2 binding-defective mutations resulted in Pho1 hyper-repression in *CTD-WT* cells and eliminated the de-repressive effect of *CTD-S7A* (S8 Fig). These mutant *ppn1* alleles were then tested, after mating and sporulation, for activity in several genetic backgrounds in which Ppn1 is essential for growth. Our initial expectations were that Ppn1 mutations that interdict the Dis2 binding site might phenocopy the synthetic lethality of *dis2*Δ with *ssu72-C13S* but not display any of the other synthetic lethal interactions characteristic of *ppn1*Δ (e.g., with *ctf1*Δ, *rhn1*Δ, *pin1*Δ, *CTD-Y1F*, *CTD-S2A*, and *CTD-T4A*) [6,14]. Whereas each of the Dis2 binding-defective alleles was indeed lethal with *ssu72-C13S*, the remarkable findings were that these *ppn1* alleles had a wider range of synthetic lethal interactions than did *dis2*Δ, whereby: (i) all of them were lethal with *pin1*Δ, *CTD-Y1F*, and *CTD-T4A*; and (ii) *501-506A* and *W510A* were lethal with *rhn1*Δ, *ctf1*Δ, and *CTD-S2A* (Fig 12). The double mutants that were viable–*ppn1-V508A ctf1*Δ, *ppn1-V508A rhn1*Δ, *ppn1-V508A CTD-S2A*, *ppn1-D515A-L516A ctf1*Δ, *ppn1- D515A-L516A rhn1*Δ, and *ppn1-D515A-L516A CTD-S2A –*were very sick at all temperatures when spot-tested for growth on YES agar (Fig 12).

### Dis2 phosphatase paralog Sds21 also interacts with Ppn1

The results in the preceding section engender the conundrum that mutating the Dis2 binding site of Ppn1 is more deleterious than the absence of Dis2. We envisioned two scenarios to account for this: (i) that Dis2 “liberated” from its contacts with Ppn1 might have negative effects on growth in the various genetic backgrounds in which the Ppn1 Dis2-box mutants are lethal; or (ii) in the absence of Dis2, another protein can interact with the Dis2-box of Ppn1 and perform some of the functions normally executed by Dis2, but this compensation is precluded by the Dis2-box mutations. If the “liberated Dis2 is bad” model is operative, then we would expect that synthetic lethality of, for example, *ppn1-W510A rhn1*Δ ought to be rescued by *dis2*Δ. However, we found, after mating and sporulation, that no viable *ppn1-W510A rhn1*Δ *dis2*Δ haploids were recovered, which militates against the “liberated Dis2 is bad” model.

**Fig 13:**
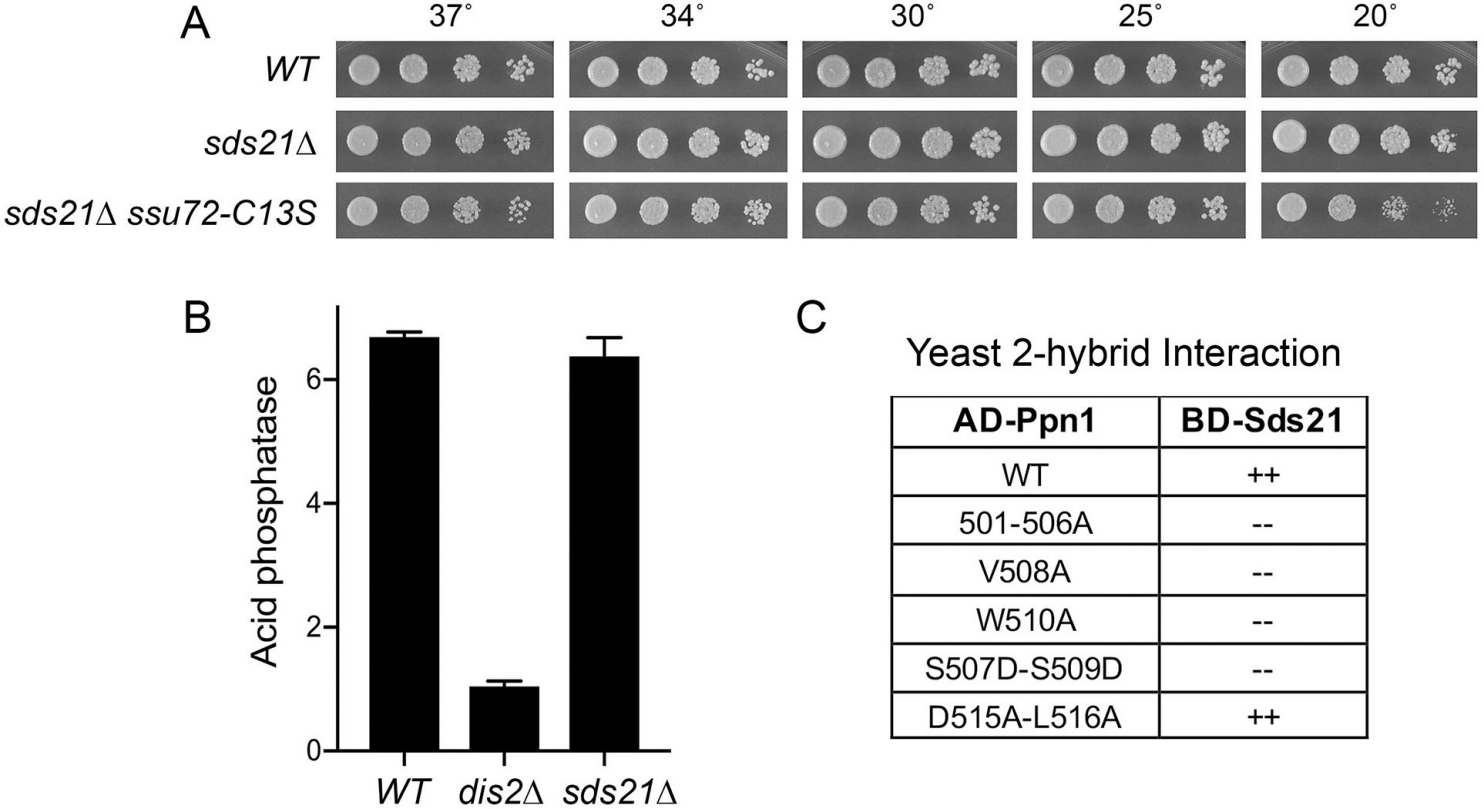
Sds21 can interact with Ppn1 via the Dis2 binding site. (A) *S*. *pombe* wild-type, *sds21*Δ, and *sds21*Δ *ssu72-C13S* strains were spot-tested for growth on YES agar at the indicated temperatures. (B) *S*. *pombe* wild-type, *dis2*Δ, and *sds21*Δ cells were grown in liquid culture at 30°C and assayed for acid phosphatase activity. (C) Full-length Ppn1 (WT and the indicated mutants) fused to the Gal4 AD were tested for 2-hybrid interactions with Gal4 BD fusions to full-length Sds21. AD/BD pairs that scored positive in both reporter assays (LacZ expression and histidine prototrophy) are indicated as ++. Pairs that were negative in both reporter assays are scored as --.

A plausible candidate to partly compensate for Dis2 deletion is its PP1 protein phosphatase paralog Sds21 [36]. Sds21, like Dis2, is inessential for fission yeast growth [36] (Fig 13A). Dis2 and Sds21 have non-identical but overlapping functions, whereby a *dis2*Δ *sds21*Δ double mutant is lethal [36]. That Sds21 does not normally play a role in CPF function when Dis2 is present is in keeping with our finding that an *sds21*Δ *ssu72-C13S* double-mutant is viable (Fig 13A) whereas *dis2*Δ is synthetically lethal with *ssu72-C13S* [6]. Moreover, deletion of Sds21 does not elicit the hyper-repression of Pho1 seen in *dis2*Δ cells (Fig 13B). Alvarez-Tabarés et al. reported that Dis2 is present in wild-type cells throughout the nucleus, whereas Sds21 is predominantly nucleolar [37]. They found that deleting Dis2 resulted in increased intracellular levels of Sds21 and detection of Sds21 at nuclear locations previously occupied by Dis2 [37]. If our conjecture that Sds21 can abet Ppn1 function in the absence of Dis2 is valid, then it should be the case that Sds21 can bind to Ppn1. A yeast 2-hybrid test affirmed that full-length Sds21 and Ppn1 interact and, moreover, that Sds21 binding was erased by Ppn1 mutations 501-506A, V508A, W510A, and S507D-S509D (Fig 13C). We conclude that Dis2 and Sds21 can interact with Ppn1 via the same Dis2-box element. Yet, the finding that the Ppn1 D515A-L516A mutation did not abolish the 2-hybrid interaction with Sds21 (Fig 13C) hints that there may be subtle differences in the Dis2-Ppn1 and Sds21-Ppn1 interfaces.

### The distinctive C-terminal tail of Dis2 is dispensable for Dis2 function

**Fig 14:**
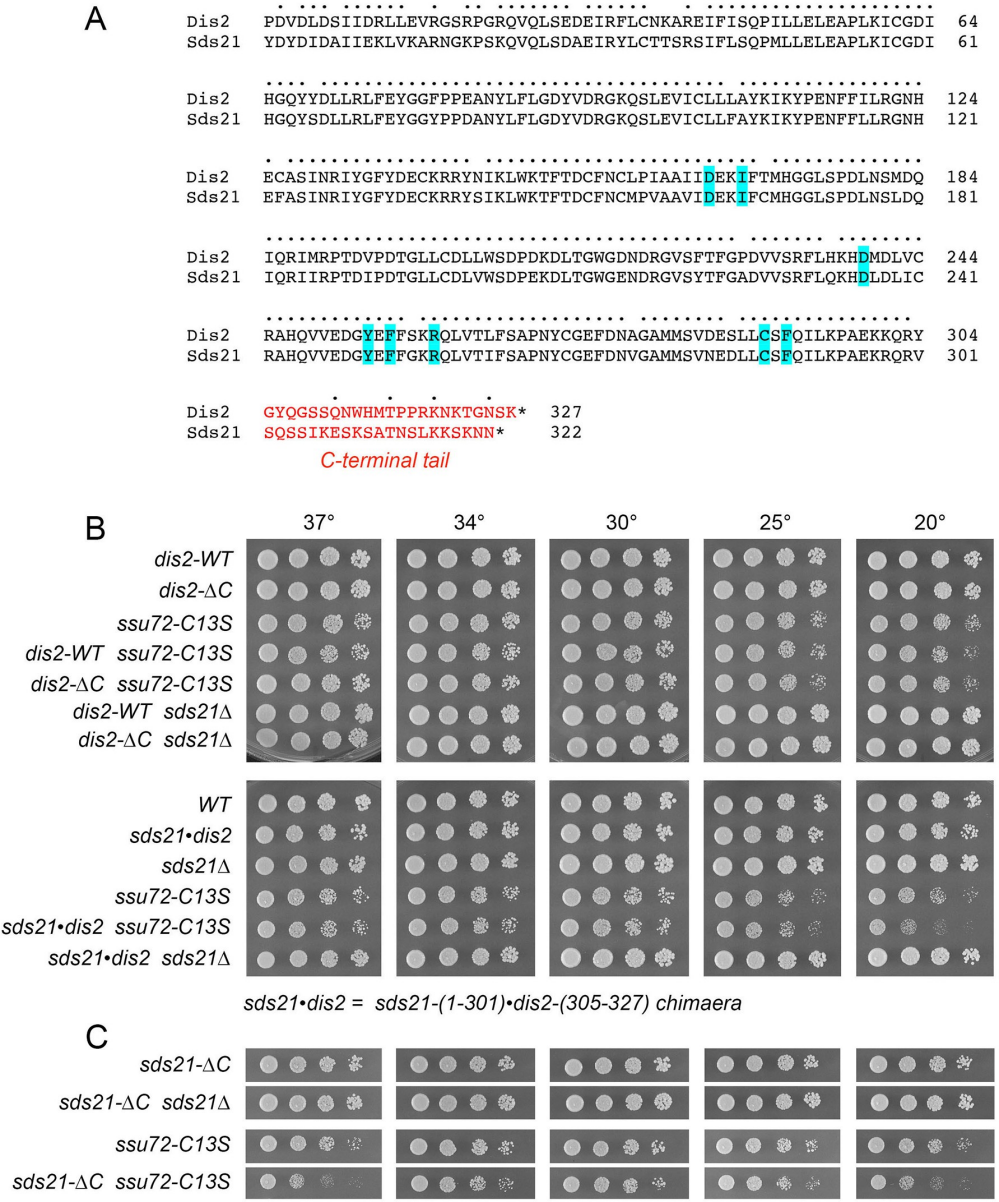
The distinctive C-terminal tail of Dis2 is dispensable for Dis2 function. (A) Primary structure alignment of Dis2 and Sds21, with positions of amino acid side chain identity/similarity indicated by dots. The divergent C-terminal tails are shown in red font. The amino acids predicted to comprise the Ppn1 binding pocket are highlighted in blue. (B and C) *S*. *pombe* strains as specified on the left were spot-tested for growth on YES agar at the indicated temperatures.

The 327-aa Dis2 and 322-aa Sds21 proteins share 252 positions of amino acid identity and 27 positions of side chain similarity (Fig 14A). Whereas Dis2 and Sds21 are nearly identical with respect to their N-terminal 304-aa and 301-aa polypeptides, the primary structures of their respective 23-aa and 21-aa C-terminal tails diverge almost completely (Fig 14A). Thr316 within the Dis2 C-terminal tail is phosphorylated in vivo, whereas the corresponding Thr313 residue in the Sds21 is not, the difference being that Dis2 Thr316 is situated within a Thr-Pro dipeptide that is targeted by cyclin-dependent protein kinases whereas the Sds21 Thr313 is flanked by an asparagine [38] (Fig 14A). Parua et al. [39] reported that Dis2 Thr316 is phosphorylated by Cdk9 and they propose that this modification diminishes Dis2’s activity as a protein phosphatase that dephosphorylates Thr-PO_4_ sites in the C-terminal repeat domain of Pol2 transcription elongation factor Spt5 as the Pol2 transcription complex encounters the cleavage/polyadenylation site (CPS) of the transcription unit. This and other studies have coalesced into a model in which Dis2 dephosphorylation of Spt5 slows Pol2 movement after traversal of the CPS and facilitates engagement of the termination machinery [13,39,40]. The impact of Thr316 phosphorylation on this scenario is unclear, insofar as Parua et al. found via PRO-seq that the phospho-ablative T316A and phosphomimetic T316D mutations behaved virtually identically to *dis2*Δ in eliciting greater read-through elongation by Pol2 to template sites downstream of the CPS [39].

To query whether the distinctive C-terminal tail of Dis2, and its phosphorylation site, contribute to Dis2 essential functions in vivo, we replaced the endogenous *dis2*^+^ gene with *dis2*-Δ*C*, which encodes Dis2-(1–304) that lacks the C-terminal tail. The *dis2*-Δ*C* strain grew as well as *dis2-WT* at all temperatures (Fig 14B). Tests of *dis2*-Δ*C* function were conducted in two genetic backgrounds–*ssu72-C13S* and *sds21*Δ–in which Dis2 is essential for growth. We obtained *dis2-*Δ*C ssu72-C13S* and *dis2-*Δ*C sds21*Δ double-mutants at the expected frequency after mating and sporulation; the double mutants grew as well as the *dis2-WT ssu72-C13S* and *dis2-WT sds21*Δ single-mutants at all temperatures (Fig 14B). We conclude that the C-terminal tail and Thr316 phosphorylation are dispensable for Dis2’s essential activities.

In light of these new results, we considered the prospect that the distinctive C-terminal tail of Sds21 might disfavor it acting in lieu of Dis2 in the context of CPF and 3’-processing (which is inessential in an otherwise wild-type background) because it directs Sds21 function elsewhere. Alternatively, the very few amino acid differences within the catalytic domains of Dis2 and Sds21 could be a decisive factor. To address the latter issue, we created a chimaeric allele, *sds21•dis2*, in which the catalytic domain of Sds21 (aa 1–301) was fused to the C-terminal tail of Dis2 (aa 305–327) and introduced *sds21•dis2* in lieu of the *dis2*^+^ chromosomal gene so that *sds21•dis2* expression is driven by the native *dis2* promoter. The salient findings were that *sds21•dis2* sufficed for growth in an *ssu72-C13S* background and in the *sds21*Δ background (Fig 14B). We surmise that: (i) there is no significant difference between the Dis2 and Sds21 catalytic domains with respect to CPF function; and (ii) swapping the Dis2 C-terminal tail into Sds21 is not deleterious.

Finally, we introduced an *sds21*-Δ*C* allele–encoding the Sds21-(1–301) catalytic domain but lacking the Sds21 C-terminal tail–in place of the *dis2*^+^ chromosomal gene so that *sds21*-Δ*C* expression is driven by the native *dis2* promoter. We found that *sds21*-Δ*C* sufficed for growth in an *ssu72-C13S* background and in the *sds21*Δ background (Fig 14C). We conclude that the catalytic domain of either Dis2 or Sds21 suffices for vegetative growth and CPF function when expressed from the *dis2* genomic locus.

### Dis2 mutations that affect interaction with Ppn1

Inspection of the structure of PNUTS in complex with PP1 (S7 Fig) highlighted interfacial PP1 amino acids Ile169, Tyr255, Phe257, Arg261, Cys291, Phe293, Asp166, and Asp240 that are conserved in fission yeast Dis2 as Ile168, Tyr254, Phe256, Arg260, Cys290, Phe292, Asp165, and Asp239. Single alanine mutations at each of these positions were introduced into the full-length BD-Dis2 expression construct and the Dis2 mutants tested for 2-hybrid interaction with full-length AD-Ppn1 (S9 Fig). Ppn1 interaction was eliminated by alanine mutations at Dis2 residues Ile168, Phe256, and Phe292 that, in PP1, comprise a hydrophobic interface with the conserved Val, Trp, and Leu moieties of PNUTS/Ppn1 (S7 Fig). Mutating interfacial Dis2 residues Tyr254 and Arg260 resulted in weaker 2-hybrid interactions in the lacZ and His reporter readouts. Mutating Dis2 Cys290 did not affect Ppn1 interaction. It was noteworthy that mutating Dis2 Asp165 and Asp239, singly or together, did not affect 2-hybrid interaction with Ppn1 (S9 Fig). These amino acids in PP1 make salt bridges to two basic amino acids in PNUTS (S7 Fig). Finally, we found that deleting the distinctive C-terminal tail of Dis2 (Fig 14A) had no impact on Ppn1 interaction in the 2-hybrid assay (S9 Fig).

### Effect of a Swd22 binding-defective mutation on Ppn1 activity in vivo

We queried whether the *R586A-Y588A-K589A* mutation (*RYK-AAA*) that selectively impaired Ppn1 binding to Swd22 in the 2-hybrid assay might mimic the synthetic lethal phenotypes of *swd22*Δ, which are identical to those of *ppn1*Δ [6]. We found that *ppn1*-(*RYK-AAA*) was lethal with *ssu72-C13S* and very sick when paired with *ctf1*Δ, *pin1*Δ, *CTD-Y1F*, or *CTD-T4A* (Fig 12).

## Discussion

Our aim in this study was to dissect the functional, physical, and genetic interactions of the fission yeast DPS (Dis2•Ppn1•Swd22) complex by focusing on its Ppn1 subunit, which mediates association of DPS with the core CPF assembly [1]. Functional output of DPS activity was gauged by transcriptional profiling of *ppn1*Δ, *swd22*Δ, and *dis2*Δ mutants. We thereby defined limited but highly concordant sets of protein-coding genes, representing ≤2% of the 5118 annotated fission yeast coding transcripts, that were up-regulated or down-regulated in *ppn1*Δ and *swd22*Δ cells (70–75% overlap). 70% of the *ppn1*Δ/*swd22*Δ up-regulated mRNAs were coordinately up in *dis2*Δ cells; 43% of the *ppn1*Δ/*swd22*Δ down-regulated mRNAs were down-regulated in *dis2*Δ cells. These transcriptomic data resonate with the DPS genetics, whereby *ppn1*Δ and *swd22*Δ display an identical spectrum of synthetic lethalities, which is broader than that of *dis2*Δ [6,7]. All three DPS null mutants are synthetically lethal with the phosphatase-dead alleles of CPF core subunit Ssu72 [6] and we see here that there was extensive overlap of the *ppn1*Δ/*swd22*Δ transcriptome with that of *ssu72-C13S* cells. The connection between the RNA-seq data and DPS function in 3’ processing and transcription termination was apparent in the case of the mRNAs of phosphate homeostasis genes *pho1* and *pho84*, which were sharply down-regulated in *DPS*Δ cells. The *PHO* genes are under repressive control by transcription of upstream flanking lncRNAs [17] and the steady-state levels of these transcripts (as gauged by Northern blot and primer extension assays), as well as cellular Pho1 activity, are hyper-repressed in *DPS*Δ and other genetic backgrounds in which 3’ processing and transcription termination are negatively affected [6,7]. A previous study that employed tiling array hybridization to analyze the transcriptome of *swd22*Δ cells indicated that 38 protein-coding transcripts were down-regulated by at least 2-fold *vis-à-vis* wild-type cells during growth at 34°C [1], of which 16 overlapped with the down-regulated set of 57 mRNAs determined here by genome-wide RNA-seq. The overlapping *swd22*Δ down-regulated genes in the microarray and RNA-seq data sets include *pho1*, *pho84*, *ecl3* (adjacent to *pho84*), and *gep4*.

Essential and inessential modules of the 710-aa Ppn1 protein were defined here by testing the effects of N- and C-terminal Ppn1 truncations in multiple genetic backgrounds in which Ppn1 is required for fission yeast growth. Ppn1 mutants were also tested for their effect on Pho1 expression, which provides a sensitive read-out of mutational effects on 3’ processing and transcription termination [6]. The N-terminal 172-aa disordered region of Ppn1 was dispensable in all assays of Ppn1 essentiality and deleting the disordered N-terminus alleviated hypomorphic phenotypes caused by deleting the C-terminal segment from aa 640–710. Assaying steady-state levels of the Ppn1 protein in the mutant strain by Western blotting with anti-Ppn1 antibody implicated the disordered N-terminus as a likely instability determinant.

We were especially interested in the functional contributions of the predicted well-folded TFIIS-like domain of Ppn1. The effects of deleting the N-terminal 330-aa segment that includes the TFIIS-like domain with respect to growth were highly dependent on the genetic background that required Ppn1 for growth. For example, loss of the TFIIS module was deleterious to *CTD-Y1F* and *CTD-S2A* cells but did not significantly affect growth of *CTD-T4A* and *pin1*Δ cells. These deleterious effects of the NΔ330 truncation were alleviated by deleting the C-terminal segment from aa 640–710 (S6 Fig). Thus, whereas the need for the Ppn1 TFIIS domain for healthy growth is contingent on the state of the Pol2 transcription and co-transcriptional RNA processing machinery and the internal domain composition of Ppn1 itself, the TFIIS domain is not strictly required for viability. However, the TFIIS domain is required for de-repression of *pho1* by CTD-S7A mutation and perturbation of IPP dynamics that elicit precocious lncRNA termination. Considering the growth properties of the ensemble of truncation mutants (S6 Fig), we can surmise that Ppn1-(386–593) represents the minimized state of the protein that can support reasonably healthy growth at 30°C in each of the genetic backgrounds tested here.

Distinct sites within Ppn1 for binding to Dis2 (spanning Ppn1 aa 506 to 532) and Swd22 (from Ppn1 aa 533 to 578) were demarcated here by yeast 2-hybrid assays. Vanoosthuyse et al. [1] had proposed that the Dis2 interaction domain of Ppn1 spans aa 501 to 555 and consists of three motifs (A, B, and C). They reported that an internally deleted version of Ppn1 (ΔABC) that lacks this region failed to interact with Dis2 in a co-IP Western assay [1]. Left untested was whether the deleted segment sufficed for Dis2 interaction; whether the ΔABC internal deletion affected Ppn1 interaction with Swd22; or whether the internal deletion disrupted Ppn1 tertiary structure. Our results define an autonomous Dis2 binding domain of Ppn1 that embraces their motifs A and B but does not include motif C. [Predicted motif C– ^550^ARVAFG^555^ –lies within the Swd22-binding domain of Ppn1 defined herein.] Via alanine-scanning of the Dis2 box of Ppn1, we identified Dis2 interaction-defective versions of full-length Ppn1 (that retained Swd22 interaction) and employed them to show that Dis2 binding is necessary for Ppn1 biological activity in multiple genetic backgrounds where Ppn1 is essential.

Intrigued by the findings that mutations in the Dis2-box of Ppn1 elicited more severe growth phenotypes than did deletion of Dis2 itself, we showed that the Dis2-box can also mediate binding to the Dis2 paralog Sds21. This led us to query the basis for the functional redundancy of Dis2 and Sds21 with respect to vegetative growth–whereby neither enzyme is essential but deletion of both is synthetically lethal–and for the seemingly unique function of Dis2 as a component of CPF that is essential for viability when the Ssu72 phosphatase subunit of CPF is inactivated. We focused on the potential role of the divergent Dis2 and Sds21 C-terminal tails as functional discriminators and found that the Dis2 C-terminal tail is dispensable and that a tail-less version of Sds21 can replace Dis2 when expressed from the *dis2* genomic locus. Thus, with respect to the genetic tests of function applied here, there is no essential role for the C-terminal tail, and the catalytic domain of either Dis2 or Sds21 suffices for function. While we do not exclude the possibility that there are other contexts or cellular pathways in which the C-terminal tails do contribute, a parsimonious interpretation and inference from our results is that differences in expression, or regulated expression, from the *dis2* versus *sds21* loci might account for the functional distinctions, e.g., if the *dis2* locus drives a significantly higher level of expression than the *sds21* locus. Indeed, that seems to be the case insofar as the results of our RNA-seq analyses of wild-type fission yeast, from two independent experiments entailing six biological replicates, show that the normalized read counts for the *dis2* mRNA transcript were 4.1-fold higher than the normalized read counts for the *sds21* transcript.

Whereas alanine scanning across the Swd22-binding domain of Ppn1 was unfruitful with respect to defining single amino acids or pairs of amino acids that were essential for Ppn1•Swd22 interaction in the 2-hybrid assay, we did obtain a triple-mutant of full-length Ppn1 that interdicted Swd22 interaction and preserved Dis2 binding. This *ppn1*-(*RYK-AAA*) allele was lethal with *ssu72-C13S* and very sick with *ctf1*Δ, *pin1*Δ, *CTD-Y1F*, or *CTD-T4A*, i.e., it did not display the full severe spectrum of synthetical lethalities associated with *swd22*Δ. It is conceivable that the Ppn1 triple mutant retained a low level of Swd22 interaction in fission yeast that sufficed for unhealthy viability in tandem with *ctf1*Δ et al.

Having made headway here in clarifying physical and genetic interactions of the DPS complex, the stage is set for a more ambitious quest for: (i) a structure of the DPS complex, potentially with an active version of the Ppn1 subunit that lacks dispensable disordered segments; and (ii) definition of the interface of the DPS complex with the CPF core assembly. There are formidable challenges involved. For example, we find that Dis2 can be abundantly expressed in bacteria but is intractably insoluble and the situation is not improved by co-expressing Dis2 in bacteria with a “biologically active” truncated version of Ppn1. Further progress will hinge on: (i) purifying DPS and CPF core complexes produced in fission yeast in quantities sufficient for biochemical and structural analyses; and (ii) delineating specific contacts between Ppn1 and CPF core (and potentially other factors involved in 3’-processing/termination), e.g., via 2-hybrid screening of a fission yeast cDNA library (fused to AD) for interactors with BD-Ppn1.

## Materials and methods

### Transcriptome profiling by RNA-seq

RNA was isolated from *S*. *pombe* wild-type, *ppn1*Δ, *swd22*Δ, and *dis2*Δ cells (6) grown in liquid YES medium at 30°C to an *A*_600_ of 0.5 to 0.6. Cells were harvested by centrifugation and total RNA was extracted via the hot phenol method. The integrity and quantity of total RNA was gauged with an Agilent Technologies 2100 Bioanalyzer. The Illumina TruSeq stranded mRNA sample preparation kit was used to purify poly(A)^+^ RNA from 500 ng of total RNA and to carry out the subsequent steps of poly(A)^+^ RNA fragmentation, strand-specific cDNA synthesis, indexing, and amplification. Indexed libraries were normalized and pooled for paired-end sequencing performed by using an Illumina NovaSeq 6000 system. FASTQ files bearing paired-end reads of length 51 bases were mapped to the *S*. *pombe* genome using HISAT2-2.1.0 with default parameters [41]. The resulting SAM files were converted to BAM files using Samtools [42]. Count files for individual replicates were generated with HTSeq-0.10.0 [43] using exon annotations from Pombase (GFF annotations, genome-version ASM294v2; source ‘ensembl’). RPKM analysis and pairwise correlations (S1 and S2 Figs) were performed as described previously [21]. Differential gene expression and fold change analysis was performed in DESeq2 [44]. Cut-off for further evaluation was set for genes that had an adjusted p-value (Benjamini-Hochberg corrected) of ≤0.05 with an average normalized count of ≥100 in either mutants or wild-type datasets, and that were up or down by at least two-fold in comparison to wild-type.

### Allelic exchange at the *ppn1* locus

Strains harboring marked wild-type and mutated *ppn1* alleles were constructed as follows. An integration cassette for wild-type *ppn1* consisted of five elements in series from 5’ to 3’: (i) a 514-bp segment of genomic DNA 5’ of the *ppn1*^*+*^ start codon; (ii) an open reading frame (ORF) encoding Ppn1; (iii) a 268-bp segment including polyA/termination signals from the *nmt1*^*+*^ gene 3’ of the *nmt1*^*+*^ stop codon, (iv) a *hygMX* gene conferring resistance to hygromycin; and (v) a 486-bp segment of genomic DNA 3’ of the *ppn1*^*+*^ stop codon. PCR with mutagenic primers was used to introduce truncation and missense mutations into the *ppn1* ORF and mutated DNA restriction fragments were inserted in the integration cassette in lieu of the wild-type *ppn1* ORF. All inserts were sequenced to exclude the presence of unwanted mutations. The integration cassettes were transfected into diploid *S*. *pombe* cells. Hygromycin-resistant transformants were selected and correct integrations at the target locus were confirmed by Southern blotting. A segment of the *ppn1*::*hygMX* allele was amplified by PCR and sequenced to verify that the desired mutations were present. The heterozygous diploids were then sporulated and hygromycin-resistant haploids were isolated.

### Mutational effects on fission yeast growth

Cultures of *S*. *pombe* strains were grown in YES liquid medium until *A*_600_ reached 0.6–0.8. The cultures were adjusted to *A*_600_ of 0.1 and 3 μl aliquots of serial 5-fold dilutions were spotted on YES agar. The plates were photographed after incubation for 2 days at 34°C, 2.5 days at 30°C and 37°C, 4 days at 25°C, and 6 days at 20°C.

### Tests of mutational synergies

Standard genetic methods were employed to generate haploid strains harboring mutations/deletions in two differently marked genes (S1 Table). In brief, pairs of haploids with truncation, missense, or null mutations were mixed on malt agar to allow mating and sporulation and then the mixture was subjected to random spore analysis. Spores (~1,500) were plated on YES agar and on media selective for marked mutant alleles; the plates were incubated at 30°C for up to 5 days to allow slow-growing progeny to germinate and form colonies. At least 500 viable progeny were screened by replica-plating for the presence of the second marker gene, or by sequentially replica-plating from YES to selective media. A finding that no haploids with both marker genes were recovered after 6 to 8 days of incubation at 30°C was taken to indicate synthetic lethality. By sequentially replica-plating and gauging the numbers of colonies at each step, we ensured that wild-type (unmarked) and the differentially marked single mutant alleles were recovered at the expected frequencies. Growth phenotypes of viable double-mutants were assessed in parallel with the individual mutants and wild-type cells at different temperatures (20°C to 37°C) as described above.

### Acid phosphatase activity

Cells were grown at 30°C in YES liquid medium. Aliquots of exponentially growing cultures were harvested, washed with water, and resuspended in water. To quantify acid phosphatase activity, reaction mixtures (200 μl) containing 100 mM sodium acetate (pH 4.2), 10 mM *p*-nitrophenyl phosphate, and cells (ranging from 0.01 to 0.1 *A*_600_ units) were incubated for 5 min at 30°C. The reactions were quenched by addition of 1 ml of 1 M sodium carbonate, the cells were removed by centrifugation, and the absorbance of the supernatant at 410 nm was measured. Acid phosphatase activity is expressed as the ratio of *A*_410_ (*p*-nitrophenol production) to *A*_600_ (cells). The data are averages (± SEM) of at least three assays using cells from three independent cultures.

### Yeast 2-hybrid assays of Ppn1 interaction with Dis2, Swd22, and Sds21

Assays were performed using reagents from Clontech as described [45]. The full-length Ppn1, Swd22, and Dis2 ORFs were cloned into pACT2.1 and pAS2-1 to generate Gal4 Activation Domain (AD) and Gal4 DNA-Binding Domain (BD) fusion proteins, respectively. The Sds21 ORF was cloned into pAS2-1. AD and BD plasmids were introduced pairwise into *S*. *cerevisiae* Y190 cells (*MATa*, *ura3-52*, *his3-200*, *ade2-101*, *lys2-801*, *trp1-901*, *leu2-3 112*, *gal4Δ*, *gal80Δ*, *cyhr*^*2*^, *LYS2*:: *GAL1*_*UAS*_*-HIS3*_*TATA*_*-HIS3*, *MEL1 URA3*:: *GAL1*_*UAS*_*-GAL1*_*TATA*_*-lacZ*) and transformants were selected for growth on SD-Trp^–^Leu^−^agar plates. X-gal assays were performed as follows. Four independent transformants were patched onto -trp-leuD plates and grown overnight at 30°C. Cells were filter-lifted from plates onto Whatman filter paper (No. 50) and immediately flash-frozen in liquid nitrogen. Filters were placed on Whatman paper (No. 3) saturated with X-gal solution (60 μM Na_2_HPO_4_, 40 μM NaH_2_PO_4_, 10 μM KCl, 1 μM MgSO_4_, 40 mM β-mercaptoethanol, 0.24 mg/mL X-gal) and incubated at 30°C for up to 6 h, then dried in a chemical hood. Cell patches that appeared blue after drying were deemed positive; those that remained white were deemed negative. Histidine prototrophy was evaluated by streaking cells to permissive (-trp-leuD), and restrictive (-trp-leu-hisD, -trp-leu-hisD + 25 mM 3AT [3-amino-1,2,4-triazole], and -trp-leu-hisD + 50 mM 3AT) conditions. Streaked cells that grew on -trp-leu-hisD + 50 mM 3AT were scored as positive for 2-hybrid interaction while those that did not grow in restrictive conditions were scored as negative.

### Anti-Ppn1 antibody

A cDNA encoding Ppn1-(173–496) was amplified by PCR and cloned into a pET-28(b)-derived vector to generate plasmid pET-His_10_Smt3-Ppn1-(173–496) for expression of the fusion protein His_10_Smt3-Ppn1-(173–496). A 1-liter culture of BL21(DE3)Codon^+^ pET-His_10_Smt3-Ppn1-(173–496) was grown at 37°C in LB medium containing 50 μg/ml kanamycin and 25 μg/ml chloramphenicol until *A*_600_ reached 0.5. The culture was then adjusted to 2% (v/v) ethanol and chilled to 4°C for 30 min. Ppn1-(173–496) expression was induced by the addition of IPTG to a final concentration of 0.5 mM and incubating the culture overnight at 17°C. Cells were harvested by centrifugation and resuspended in lysis buffer containing 500 mM NaCl, 50 mM Tris-HCl pH 8.0, 10% glycerol, 10 mM imidazole, 0.5 mM PMSF. All subsequent purification procedures were performed at 4°C. Cell lysis was achieved by adding lysozyme to 0.2 mg/ml and incubation for 1 h, followed by sonication to reduce viscosity. The lysate was centrifuged at 40,000g for 45 min and the supernatant was applied to a 5-ml Ni-NTA-agarose column that had been equilibrated in lysis buffer. The resin was washed twice with 50 ml of lysis buffer containing 20 mM imidazole. His_10_Smt3-Ppn1-(173–496) was eluted with lysis buffer containing 300 mM imidazole. The His_10_Smt3 tag was cleaved by treatment with Ulp1 protease during overnight dialysis against lysis buffer. Ppn1-(173–496) was separated from His_10_Smt3 by a second round of Ni-affinity chromatography, during which Ppn1-(173–496) was recovered in the flow-through fraction. Purified Ppn1-(173–496) was concentrated by centrifugal ultrafiltration to 2 mg/ml.

Rabbit immunization with purified Ppn1-(173–496) and preparation of antiserum were performed by Pocono Hills Rabbit Farm and Laboratory (Canadensis, PA) according to their 70 Day Antibody Production Protocol. Anti-Ppn1 antibody was purified from 5 ml of rabbit serum by affinity chromatography as follows. 2 mg of purified Ppn1-(173–496) was dialyzed against coupling buffer (500 mM NaCl, 50 mM HEPES pH 7.9, 5% glycerol) and then coupled to 3 ml of Affigel-10 resin (BioRad) by incubation overnight at 4°C. The resin was washed serially with 100 mM Tris-HCl (pH 7.4); 200 mM glycine (pH 2.6); 1 M Tris-HCl (pH 7.4); and 150 mM NaCl in 20 mM Tris-HCl (pH 7.4). The antigen-coupled resin was then mixed with 5 ml of rabbit immune serum overnight at 4°C on a nutator. The resin was poured into a column and washed thoroughly with TBS (150 mM NaCl, 10 mM Tris-HCl pH 7.5) until no further protein was eluted. Bound antibodies were then eluted with 200 mM glycine (pH 2.6). Fractions (0.5 ml) were collected in tubes containing 50 μl of 1 M Tris-HCl (pH 8.3) to adjust to pH 7.5. Protein-containing eluate fractions were pooled and dialyzed against buffer containing 50 mM NaCl, 200 mM Tris-HCl (pH 7.5), 0.1 mM EDTA, 0.1 mM β-mercaptoethanol, 5% glycerol.

### Western blotting

Fission yeast cells were grown at 30°C in liquid YES medium until *A*_600_ reached 0.6 to 0.8. Aliquots (10 ml) of cells were collected by centrifugation and washed with cold 20% trichloroacetic acid (TCA) and washed cell pellets were frozen at –80°C. The cells were resuspended in cold 20% TCA and disrupted by vortexing with glass beads for 3 min at 4°C. Cellular material was separated from beads by low-speed centrifugation, and additional material was recovered by washing beads with one volume 5% TCA. Cellular material was supplemented with one volume of 5% TCA and precipitated by high-speed centrifugation. The precipitate was washed with ice-cold ethanol and resuspended in 1 M Tris-HCl, pH 8.0. Protein concentration was measured according to absorbance at 280 nm using a NanoDrop 2000 spectrophotometer. Samples were heated for 5 min at 95°C in SDS buffer and then resolved by electrophoresis through a 10% polyacrylamide gel containing 0.1% SDS. Polypeptides were transferred to PVDF membranes by using an iBlot2 apparatus (Invitrogen). Membranes were washed three times with TBST (10 mM Tris-HCl, pH 7.5, 150 mM NaCl, 0.1% Tween 20), then blocked for 16 h with 5% milk in TBST. Primary hybridization was performed with affinity-purified anti-Ppn1 diluted 1:2000 in 5% milk in TBST for 1 h at 22°C. Membranes were again washed three times with TBST. Secondary hybridization was performed with HRP-conjugated anti-Rabbit IgG (produced in donkey, GE Healthcare) diluted 1:10000 in 5% milk in TBST for 1 h at 22°C. Membranes were again washed three times with TBST. Chemiluminescence was induced by incubating membranes with freshly mixed Western Blot detection reagent (GE Healthcare) for 1 min. Membranes were immediately exposed to BioMax x-ray film (Kodak).

## Supporting information

S1 Fig RNA-seq read counts for triplicate biological replicates. (PDF)

S2 Fig RNA-seq data reproducibility between biological replicates. (PDF)

S3 Fig Predicted structural homology of Ppn1-(186–325) to the TFIIS-like domain of PNUTS. (Top panel) Stereo view of the Phyre2 homology model of Ppn1 templated on the fold of the transcription elongation factor TFIIS-like N-terminal domain of rat PNUTS (pdb 6VTI). (Bottom panel) Primary structure alignment of the homologous TFIIS-like domains of Ppn1 and PNUTS generated by Phyre2. Positions of amino acids identity and sidechain similarity are denoted by • above the alignment.
(PDF)

S4 Fig Western blot of Ppn1 N-terminal truncation mutants. Whole-cell extracts from wild-type *ppn1-(1–710)*, *ppn1*Δ, and the indicated *ppn1* truncation strains growing logarithmically at 30°C were resolved by SDS-PAGE and subjected to Western blotting with polyclonal Ppn1 antibodies. The positions and sizes (in kilodaltons) of marker polypeptides are indicated at left.
(PDF)

S5 Fig Western blot of Ppn1 missense and C-terminal truncation mutants. Whole-cell extracts from wild-type *ppn1-(1–710)*, *ppn1*Δ, and the indicated *ppn1* mutant strains growing logarithmically at 30°C were resolved by SDS-PAGE and subjected to Western blotting with polyclonal Ppn1 antibodies. The full-length wild-type (WT) and C-terminally truncated Ppn1 polypeptides are denoted by dots. The position of a 100 kDa marker polypeptide is shown on the left.
(PDF)

S6 Fig Summary of growth effects of ppn1 truncations in different genetic backgrounds. *ppn1* mutants specified in the top rows were crossed to the mutant strains specified in the left-most column. Synthetically lethal pairs of alleles are highlighted in red boxes. The yellow boxes indicate synthetic growth defects (sick or very sick). Viable double mutants without a synthetic defect are indicated by a plain green box. Viable double mutants that displayed temperature-sensitive (*ts*) or cold-sensitive (*cs*) defects are annotated as such in its green box.
(PDF)

S7 Fig Conservation of PP1/Dis2 binding motifs in PNUTS and Ppn1. Stereo view of the interface of PNUTS (colored cyan) with PP1C (colored green) from the 2.2 Å crystal structure (pdb 4MOY). Interfacial amino acid side chains are rendered as stick models. Salt bridges are depicted as black dashed lines. Van der Waals contacts are shown as magenta dashed lines. The two manganese ions in the phosphatase active site are magenta spheres; a phosphate anion in the active site is rendered as a stick model. The amino acid sequences of the PP1/Dis2 binding motifs in PNUTS and Ppn1 are aligned at bottom. The conserved hydrophobic interfacial amino acids–Val, Trp, and Leu–are in white font on black background. The two basic side chains that form salt bridges to the phosphatase are underlined.
(PDF)

S8 Fig Ppn1 mutations defective for Dis2 or Swd22 binding hyper-repress Pho1 expression. *S*. *pombe CTD-WT* or *CTD-S7A* strains bearing the indicated *ppn1* alleles were grown in liquid culture at 30°C and assayed for acid phosphatase activity.
(PDF)

S9 Fig Dis2 mutations that affect interaction with Ppn1. Gal4 BD fusions to full-length Dis2 alanine mutants and the C-terminal deletion mutant Dis2-(1–304) were tested for 2-hybrid interactions with full-length Ppn1 fused to the Gal4 AD. BD/AD pairs that scored positive in both reporter assays (LacZ expression and histidine prototrophy) are indicated as ++. Pairs that were negative in both reporter assays are scored as—. Weakly positive pairs are scored as ±±.
(PDF)

S1 Table Fission yeast strains used in this study. (PDF)
