# Supplementary material for: Structure-function analysis of fission yeast cleavage and polyadenylation factor (CPF) subunit Ppn1 and its interactions with Dis2 and Swd22
Source: PLoS Genet. 2021 Mar 12;17(3):e1009452. doi: 10.1371/journal.pgen.1009452 (PMC7990198; doi:10.1371/journal.pgen.1009452)
Supplement: S1 Fig — (PDF) [file pgen.1009452.s001.pdf]

| Sample | Total Paired Reads | Mapped Reads |
|---|---|---|
| WT (1) | 26,820,778 | 25,069,976 (93%) |
| WT (2) | 26,799,612 | 24,896,074 (93%) |
| WT (3) | 24,623,533 | 22,981,899 (93%) |
| *ppn1*Δ (1) | 27,074,925 | 25,654,708 (95%) |
| *ppn1*Δ (2) | 21,792,674 | 20,210,444 (93%) |
| *ppn1*Δ (3) | 28,302,106 | 26,637,590 (94%) |
| *swd22*Δ (1) | 24,435,616 | 23,082,133 (94%) |
| *swd22*Δ (2) | 24,699,951 | 23,577,405 (95%) |
| *swd22*Δ (3) | 23,445,504 | 22,211,684 (95%) |
| *dis2*Δ (1) | 26,747,724 | 25,468,701 (95%) |
| *dis2*Δ (2) | 23,488,584 | 22,144,361 (94%) |
| *dis2*Δ (3) | 24,673,733 | 23,340,878 (95%) |

S1 Fig. RNA-seq read counts for triplicate biological replicates.
