# Supplementary material for: Structure-function analysis of fission yeast cleavage and polyadenylation factor (CPF) subunit Ppn1 and its interactions with Dis2 and Swd22
Source: PLoS Genet. 2021 Mar 12;17(3):e1009452. doi: 10.1371/journal.pgen.1009452 (PMC7990198; doi:10.1371/journal.pgen.1009452)
Supplement: S2 Fig — (PDF) [file pgen.1009452.s002.pdf]

Sample pairs	Pearson Coefficient
WT (1) vs (2)	0.987
WT (2) vs (3)	0.987
WT (1) vs (3)	0.987
*ppn1*Δ (1) vs (2)	0.985
*ppn1*Δ (2) vs (3)	0.983
*ppn1*Δ (1) vs (3)	0.988
*swd22*Δ (1) vs (2)	0.984
*swd22*Δ (2) vs (3)	0.983
*swd22*Δ (1) vs (3)	0.987
*dis2*Δ (1) vs (2)	0.987
*dis2*Δ (2) vs (3)	0.987
*dis2*Δ (1) vs (3)	0.986

S2 Fig. RNA-seq data reproducibility between biological replicates.
