# Supplementary material for: Structure-function analysis of fission yeast cleavage and polyadenylation factor (CPF) subunit Ppn1 and its interactions with Dis2 and Swd22
Source: PLoS Genet. 2021 Mar 12;17(3):e1009452. doi: 10.1371/journal.pgen.1009452 (PMC7990198; doi:10.1371/journal.pgen.1009452)
Supplement: S3 Fig — (Top panel) Stereo view of the Phyre2 homology model of Ppn1 templated on the fold of the transcription elongation factor TFIIS-like N-terminal domain of rat PNUTS (pdb 6VTI). (Bottom panel) Primary structure alignment of the homologous TFIIS-like domains of Ppn1 and PNUTS generated by Phyre2. Positions of amino acids identity and sidechain similarity are denoted by • above the alignment. (PDF) [file pgen.1009452.s003.pdf]

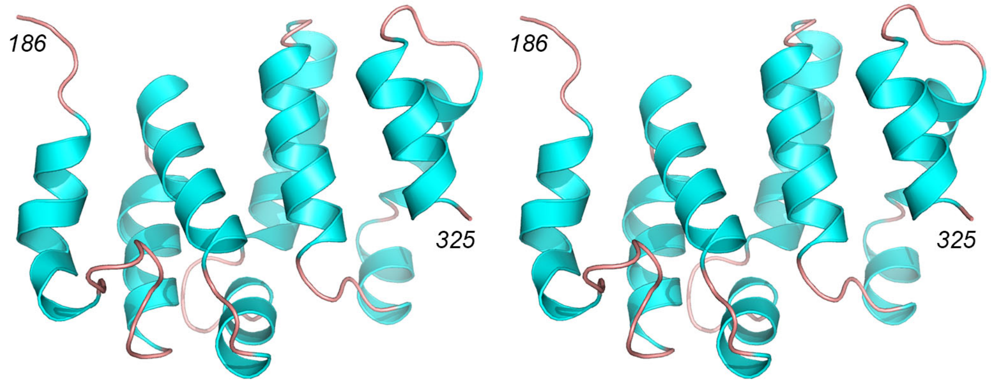


```
             .. . ..  .   .     .      . .   ..        .  .....
Ppn1      SSSDPLEDLRLTLTECLNPINIVQAPKECAAILVNLMSNITQDDQKLVFLDLLKSKVGNS 245
PNUTS     GPIDPKELLK-GLDSFLTRDGEVKSVDGIAKI-FSLMKEARKMVSRCTYLNIILQTRAPE  61

          .    .. .       .  .           ..  ... .  .... . . .    . .
Ppn1      IYSQLVDGGRKLFLPKLRNWFVSAIRSKHDELIHLILLVLANLPLTTEKLAEVKFGKPIL 305
PNUTS     VLVKFIDVGG---YKLLNSWLTYSKTTNNIPLLQQILLTLQHLPLTVDHLKQNNTAKLVK 118

           . . . .  .. ..  .
Ppn1      IVKKKSTNSVIRQLAENLSE  325
PNUTS     QLSKSSEDEELRKLASVLVS  138
```

S3 Fig. Predicted structural homology of Ppn1-(186-325) to the TFIIS-like domain of PNUTS. (Top panel) Stereo view of the Phyre2 homology model of Ppn1 templated on the fold of the transcription elongation factor TFIIS-like N-terminal domain of rat PNUTS (pdb 6VTI). (Bottom panel) Primary structure alignment of the homologous TFIIS-like domains of Ppn1 and PNUTS generated by Phyre2. Positions of amino acids identity and sidechain similarity are denoted by • above the alignment.
