# Supplementary material for: Structure-function analysis of fission yeast cleavage and polyadenylation factor (CPF) subunit Ppn1 and its interactions with Dis2 and Swd22
Source: PLoS Genet. 2021 Mar 12;17(3):e1009452. doi: 10.1371/journal.pgen.1009452 (PMC7990198; doi:10.1371/journal.pgen.1009452)
Supplement: S4 Fig — Whole-cell extracts from wild-type ppn1-(1–710), ppn1Δ, and the indicated ppn1 truncation strains growing logarithmically at 30°C were resolved by SDS-PAGE and subjected to Western blotting with polyclonal Ppn1 antibodies. The positions and sizes (in kilodaltons) of marker polypeptides are indicated at left. (PDF) [file pgen.1009452.s004.pdf]

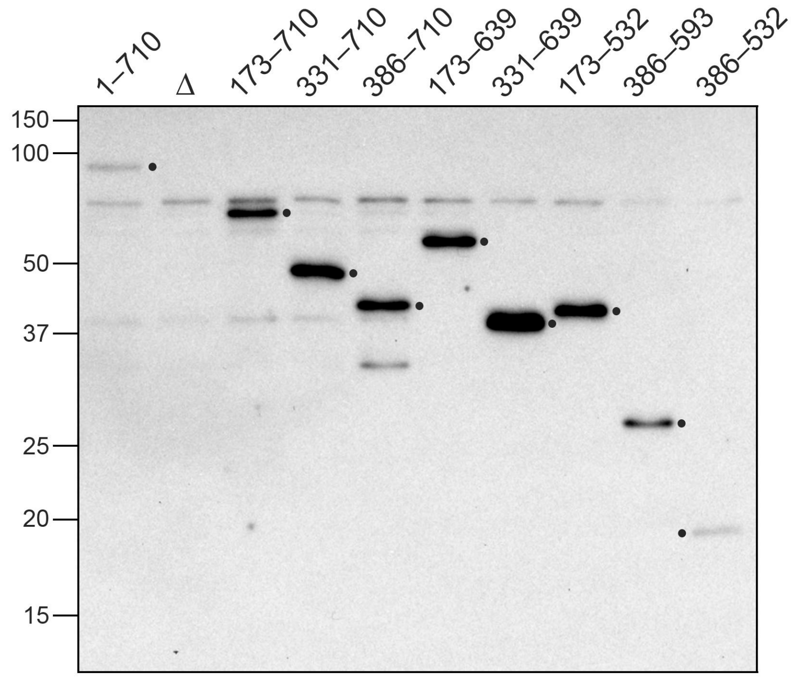

S4 Fig. Western blot of Ppn1 N-terminal truncation mutants. Whole-cell extracts from wild-type *ppn1-(1-710), ppn1*Δ, and the indicated *ppn1* truncation strains growing logarithmically at 30˚C were resolved by SDS-PAGE and subjected to Western blotting with polyclonal Ppn1 antibodies. The positions and sizes (in kilodaltons) of marker polypeptides are indicated at left.
