# Supplementary material for: Structure-function analysis of fission yeast cleavage and polyadenylation factor (CPF) subunit Ppn1 and its interactions with Dis2 and Swd22
Source: PLoS Genet. 2021 Mar 12;17(3):e1009452. doi: 10.1371/journal.pgen.1009452 (PMC7990198; doi:10.1371/journal.pgen.1009452)
Supplement: S5 Fig — Whole-cell extracts from wild-type ppn1-(1–710), ppn1Δ, and the indicated ppn1 mutant strains growing logarithmically at 30°C were resolved by SDS-PAGE and subjected to Western blotting with polyclonal Ppn1 antibodies. The full-length wild-type (WT) and C-terminally truncated Ppn1 polypeptides are denoted by dots. The position of a 100 kDa marker polypeptide is shown on the left. (PDF) [file pgen.1009452.s005.pdf]

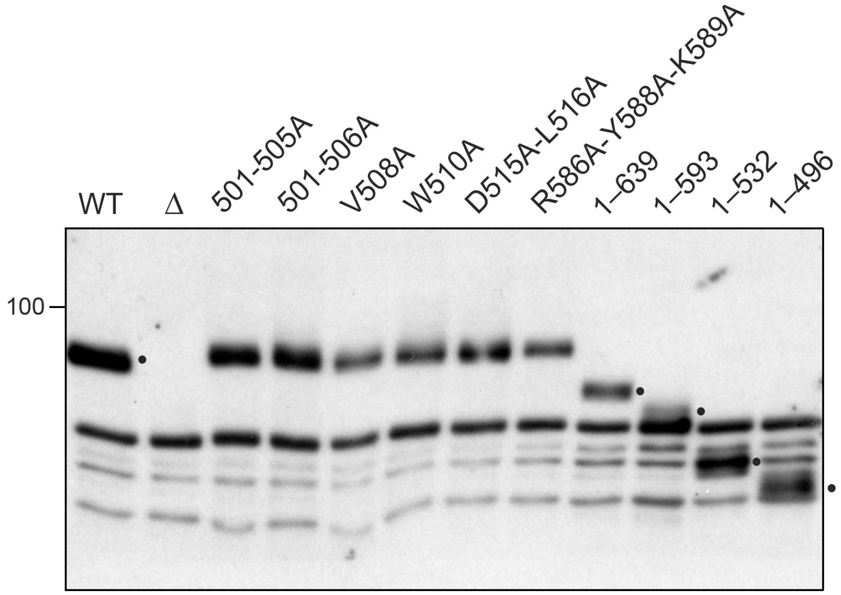

S5 Fig. Western blot of Ppn1 missense and C-terminal truncation mutants. Whole-cell extracts from wild-type *ppn1-(1-710), ppn1*Δ, and the indicated *ppn1* mutant strains growing logarithmically at 30˚C were resolved by SDS-PAGE and subjected to Western blotting with polyclonal Ppn1 antibodies. The full-length wild-type (WT) and C-terminally truncated Ppn1 polypeptides are denoted by dots. The position of a 100 kDa marker polypeptide is shown on the left.
