# Supplementary material for: Structure-function analysis of fission yeast cleavage and polyadenylation factor (CPF) subunit Ppn1 and its interactions with Dis2 and Swd22
Source: PLoS Genet. 2021 Mar 12;17(3):e1009452. doi: 10.1371/journal.pgen.1009452 (PMC7990198; doi:10.1371/journal.pgen.1009452)
Supplement: S6 Fig — ppn1 mutants specified in the top rows were crossed to the mutant strains specified in the left-most column. Synthetically lethal pairs of alleles are highlighted in red boxes. The yellow boxes indicate synthetic growth defects (sick or very sick). Viable double mutants without a synthetic defect are indicated by a plain green box. Viable double mutants that displayed temperature-sensitive (ts) or cold-sensitive (cs) defects are annotated as such in its green box. (PDF) [file pgen.1009452.s006.pdf]

N-terminal truncations

	ppn1Δ	*173-710*	*331-710*	*386-710*	*451-710*	*488-710*
ctf1Δ	Lethal		cs	cs	very sick	Lethal
rhn1Δ	Lethal			ts	ts	ts
ssu72-C13S	Lethal		ts	ts	Lethal	Lethal
pin1Δ	Lethal		sick	sick	very sick	Lethal
CTD-Y1F	Lethal		sick	sick	very sick	very sick
CTD-S2A	Lethal		sick	sick	sick	very sick
CTD-T4A	Lethal		cs	cs	sick	very sick

C-terminal truncations

	ppn1Δ	*1-639*	*1-606*	*1-593*	*1-532*	*1-496*
ctf1Δ	Lethal	cs/ts	cs/ts	ts	Lethal	Lethal
rhn1Δ	Lethal	ts	ts	ts	Lethal	Lethal
ssu72-C13S	Lethal	sick	sick	very sick	Lethal	Lethal
pin1Δ	Lethal	ts	ts	ts	Lethal	Lethal
CTD-Y1F	Lethal	very sick	very sick	very sick	Lethal	Lethal
CTD-S2A	Lethal	cs/ts	cs/ts	sick	Lethal	Lethal
CTD-T4A	Lethal	ts	ts	cs/ts	Lethal	Lethal

N/C-terminal truncations

	ppn1Δ	*173-639*	*331-639*	*386-593*	*173-532*	*386-532*
ctf1Δ	Lethal		cs		cs, ts	cs
rhn1Δ	Lethal				ts	ts
ssu72-C13S	Lethal				Lethal	very sick
pin1Δ	Lethal				Lethal	very sick
CTD-Y1F	Lethal		cs	cs	very sick	sick
CTD-S2A	Lethal		cs	cs	sick	cs
CTD-T4A	Lethal		cs	cs	very sick	very sick

S6 Fig. Summary of growth effects of *ppn1* truncations in different genetic backgrounds. *ppn1* mutants specified in the top rows were crossed to the mutant strains specified in the left-most column. Synthetically lethal pairs of alleles are highlighted in red boxes. The yellow boxes indicate synthetic growth defects (sick or very sick). Viable double mutants without a synthetic defect are indicated by a plain green box. Viable double mutants that displayed temperature-sensitive (*ts*) or cold-sensitive (*cs*) defects are annotated as such in its green box.
