# Supplementary material for: Structure-function analysis of fission yeast cleavage and polyadenylation factor (CPF) subunit Ppn1 and its interactions with Dis2 and Swd22
Source: PLoS Genet. 2021 Mar 12;17(3):e1009452. doi: 10.1371/journal.pgen.1009452 (PMC7990198; doi:10.1371/journal.pgen.1009452)
Supplement: S7 Fig — Stereo view of the interface of PNUTS (colored cyan) with PP1C (colored green) from the 2.2 Å crystal structure (pdb 4MOY). Interfacial amino acid side chains are rendered as stick models. Salt bridges are depicted as black dashed lines. Van der Waals contacts are shown as magenta dashed lines. The two manganese ions in the phosphatase active site are magenta spheres; a phosphate anion in the active site is rendered as a stick model. The amino acid sequences of the PP1/Dis2 binding motifs in PNUTS and Ppn1 are aligned at bottom. The conserved hydrophobic interfacial amino acids–Val, Trp, and Leu–are in white font on black background. The two basic side chains that form salt bridges to the phosphatase are underlined. (PDF) [file pgen.1009452.s007.pdf]

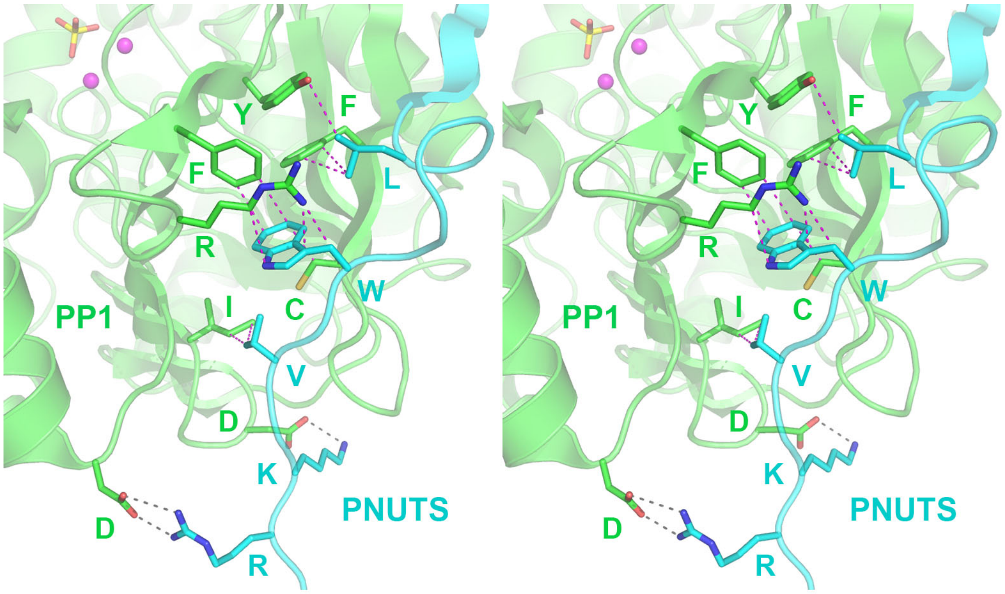


```
Ppn1     KRKKKSVSWKPDNDLVQV
PNUTS    GRKRKTVTWPEEGKLREY
```

S7 Fig. Conservation of PP1/Dis2 binding motifs in PNUTS and Ppn1. Stereo view of the interface of PNUTS (colored cyan) with PP1C (colored green) from the 2.2 Å crystal structure (pdb 4MOY). Interfacial amino acid side chains are rendered as stick models. Salt bridges are depicted as black dashed lines. Van der Waals contacts are shown as magenta dashed lines. The two manganese ions in the phosphatase active site are magenta spheres; a phosphate anion in the active site is rendered as a stick model. The amino acid sequences of the PP1/Dis2 binding motifs in PNUTS and Ppn1 are aligned at bottom. The conserved hydrophobic interfacial amino acids – Val, Trp, and Leu – are in white font on black background. The two basic side chains that form salt bridges to the phosphatase are underlined.
