# Supplementary material for: Structure-function analysis of fission yeast cleavage and polyadenylation factor (CPF) subunit Ppn1 and its interactions with Dis2 and Swd22
Source: PLoS Genet. 2021 Mar 12;17(3):e1009452. doi: 10.1371/journal.pgen.1009452 (PMC7990198; doi:10.1371/journal.pgen.1009452)
Supplement: S8 Fig — S. pombe CTD-WT or CTD-S7A strains bearing the indicated ppn1 alleles were grown in liquid culture at 30°C and assayed for acid phosphatase activity. (PDF) [file pgen.1009452.s008.pdf]

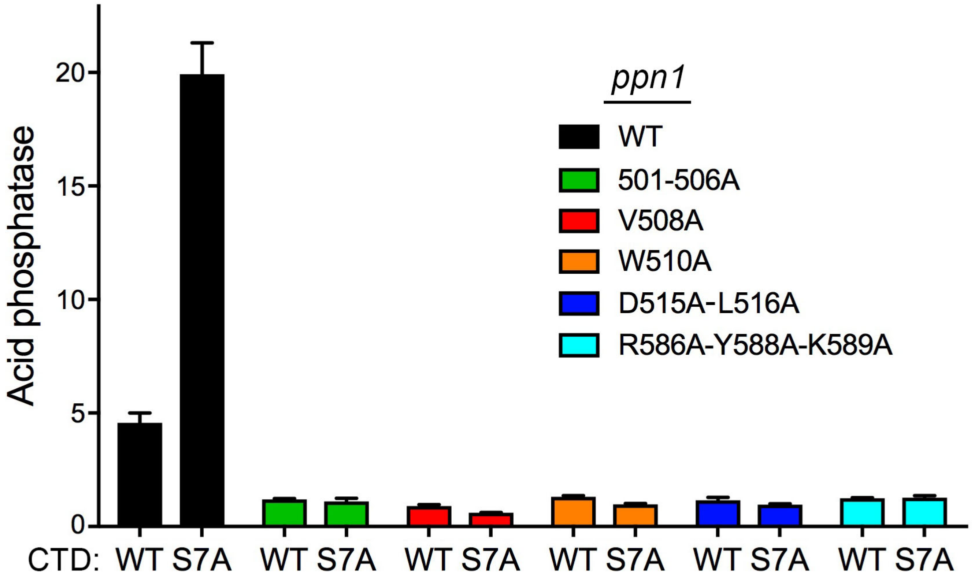

S8 Fig. Ppn1 mutations defective for Dis2 or Swd22 binding hyper-repress Pho1 expression. *S. pombe CTD-WT* or *CTD-S7A* strains bearing the indicated *ppn1* alleles were grown in liquid culture at 30°C and assayed for acid phosphatase activity.
