# Supplementary material for: Structure-function analysis of fission yeast cleavage and polyadenylation factor (CPF) subunit Ppn1 and its interactions with Dis2 and Swd22
Source: PLoS Genet. 2021 Mar 12;17(3):e1009452. doi: 10.1371/journal.pgen.1009452 (PMC7990198; doi:10.1371/journal.pgen.1009452)
Supplement: S9 Fig — Gal4 BD fusions to full-length Dis2 alanine mutants and the C-terminal deletion mutant Dis2-(1–304) were tested for 2-hybrid interactions with full-length Ppn1 fused to the Gal4 AD. BD/AD pairs that scored positive in both reporter assays (LacZ expression and histidine prototrophy) are indicated as ++. Pairs that were negative in both reporter assays are scored as—. Weakly positive pairs are scored as ±±. (PDF) [file pgen.1009452.s009.pdf]

BD-Dis2	AD-Ppn1
I168A	--
Y254A	±±
F256A	--
R260A	±±
C290A	++
F292A	--
D165A	++
D239A	++
D165A-D239A	++
ΔC	++

S9 Fig. Dis2 mutations that affect interaction with Ppn1. Gal4 BD fusions to full-length Dis2 alanine mutants and the C-terminal deletion mutant Dis2-(1-304) were tested for 2-hybrid interactions with full-length Ppn1 fused to the Gal4 AD. BD/AD pairs that scored positive in both reporter assays (LacZ expression and histidine prototrophy) are indicated as ++. Pairs that were negative in both reporter assays are scored as --. Weakly positive pairs are scored as ±±.
