# Supplementary material for: Structure-function analysis of fission yeast cleavage and polyadenylation factor (CPF) subunit Ppn1 and its interactions with Dis2 and Swd22
Source: PLoS Genet. 2021 Mar 12;17(3):e1009452. doi: 10.1371/journal.pgen.1009452 (PMC7990198; doi:10.1371/journal.pgen.1009452)
Supplement: S1 Table — (PDF) [file pgen.1009452.s010.pdf]

S1 Table: Fission yeast strains used in this study		
Name	Relevant Genotype	Source
JS77 (parental strain)	*ade6-m216 leu1-32 ura4-D18 his3-D1 (h–)*	[1]
JS78 (parental strain)	*ade6-m210 leu1-32 ura4-D18 his3-D1 (h+)*	[1]
AS 2387	*ppn1Δ::hygMX*	This study
AS 2428	*ppn1-(81-710)::hygMX*	This study
AS 2388	*ppn1-(173-710)::hygMX*	This study
AS 2463	*ppn1-(331-710)::hygMX*	This study
AS 2554	*ppn1-(386-710)::hygMX*	This study
AS 2555	*ppn1-(451-710)::hygMX*	This study
AS 2556	*ppn1-(488-710)::hygMX*	This study
AS 2390	*ppn1-(1-639)::hygMX*	This study
AS 2389	*ppn1-(1-606)::hygMX*	This study
AS 2464	*ppn1-(1-593)::hygMX*	This study
AS 2557	*ppn1-(1-532)::hygMX*	This study
AS 2465	*ppn1-(1-496)::hygMX*	This study
AS 2558	*ppn1-(173-639)::hygMX*	This study
AS 2559	*ppn1-(331-639)::hygMX*	This study
BB0003	*ppn1-(173-532)::hygMX*	This study
BB0001	*ppn1-(386-593)::hygMX*	This study
BB0002	*ppn1-(386-532)::hygMX*	This study
BB0004	*ppn1-(501-506A)::hygMX*	This study
BB0048	*ppn1-V508A::hygMX*	This study
BB0050	*ppn1-W510A::hygMX*	This study
BB0052	*ppn1-(D515A L516A)::hygMX*	This study
BB0054	*ppn1-(R586A Y588A K589A)::hygMX*	This study
BB0109	*rpb1-CTD-Y1F::natMX*	[2]
BB0110	*rpb1-CTD-S2A::natMX*	[3]
BB0111	*rpb1-CTD-T4A::natMX*	[2]
BB0112	*rpb1-CTD-S7A::natMX*	[2]
AS 2218	*dis2Δ::natMX*	[2]
AS 2020	*dis2Δ::ura4*$^{+}$	[4]
BB0113	*sds21Δ::ura4*$^{+}$	[4]
BB0114	*ctf1Δ::kanMX*	[2]
BB0115	*rhn1Δ::kanMX*	[2]
BB0116	*ssu72-C13S::kanMX*	[5]
AS612	*ssu72-C13S::natMX*	[2]
BB0117	*pin1Δ::kanMX*	[6]
BS286	*dis2-ΔC::kanMX*	This study
BS350	*dis2-(sds21-dis2)::kanMX*	This study
BS467	*dis2-(sds21-ΔC)::natMX*	This study

References

1. Pei Y, Du H, Singer J, Stamour C, Granitto S, Shuman S, et al. Cyclin-dependent kinase 9 (Cdk9) of fission yeast is activated by the CDK-activating kinase Csk1, overlaps functionally with the TFIIH-associated kinase Mcs6, and associates with the mRNA cap methyltransferase Pcm1 in vivo. Mol Cell Biol. 2006; 26:777-788.
2. Sanchez AM, Shuman S, Schwer B. RNA polymerase II CTD interactome with 3' processing and termination factors in fission yeast and its impact on phosphate homeostasis. Proc Natl Acad Sci USA. 2018; 115:E10652-E10661.
3. Schwer B, Bitton DA, Sanchez AM, Bähler J, Shuman S. Individual letters of the RNA polymerase II CTD code govern distinct gene expression programs in fission yeast. Proc Natl Acad Sci USA. 2014; 111:4185-4190.
4. Parua PK, Booth GT, Sansó M, Benjamin B, Tanny JC, Lis JT, et al. A Cdk9-PP1 switch regulates the elongation-termination transition of RNA polymerase II. Nature. 2018; 558:460-464.
5. Schwer B, Sanchez AM, Shuman S. RNA polymerase II CTD phospho-sites Ser5 and Ser7 govern phosphate homeostasis in fission yeast. RNA. 2015; 21:1770-1780.
6. Sanchez AM, Garg A, Shuman S, Schwer B. Genetic interactions and transcriptomics implicate fission yeast CTD prolyl isomerase Pin1 as an agent of RNA 3' processing and transcription termination that functions via its effects on CTD phosphatase Ssu72. Nucleic Acids Res. 2020; 48:4811-4826.
